# Pattern Recognition of the Multiple Sclerosis Syndrome

**DOI:** 10.3390/brainsci7100138

**Published:** 2017-10-24

**Authors:** Rana K. Zabad, Renee Stewart, Kathleen M. Healey

**Affiliations:** 1 Department of Neurological Sciences, University of Nebraska Medical Center College of Medicine, Omaha, NE 68198-8440, USA; khealey@unmc.edu; 2 University of Nebraska Medical Center College of Nursing, Omaha, NE 68198-5330, USA; renee.stewart@unmc.edu

**Keywords:** MS, NMOSD, clinically isolated syndrome (CIS), optic neuritis, transverse myelitis, brainstem syndrome, tumefactive demyelinating lesions, AQP4 antibodies, MOG antibodies

## Abstract

During recent decades, the autoimmune disease neuromyelitis optica spectrum disorder (NMOSD), once broadly classified under the umbrella of multiple sclerosis (MS), has been extended to include autoimmune inflammatory conditions of the central nervous system (CNS), which are now diagnosable with serum serological tests. These antibody-mediated inflammatory diseases of the CNS share a clinical presentation to MS. A number of practical learning points emerge in this review, which is geared toward the pattern recognition of optic neuritis, transverse myelitis, brainstem/cerebellar and hemispheric tumefactive demyelinating lesion (TDL)-associated MS, aquaporin-4-antibody and myelin oligodendrocyte glycoprotein (MOG)-antibody NMOSD, overlap syndrome, and some yet-to-be-defined/classified demyelinating disease, all unspecifically labeled under *MS syndrome*. The goal of this review is to increase clinicians’ awareness of the clinical nuances of the autoimmune conditions for MS and NMSOD, and to highlight highly suggestive patterns of clinical, paraclinical or imaging presentations in order to improve differentiation. With overlay in clinical manifestations between MS and NMOSD, magnetic resonance imaging (MRI) of the brain, orbits and spinal cord, serology, and most importantly, high index of suspicion based on pattern recognition, will help lead to the final diagnosis.

## 1. Introduction

A multiple sclerosis (MS) diagnosis is at the forefront when a woman or man aged 20–50 years presents neurological symptoms and/or white matter lesions on magnetic resonance imaging (MRI) of the brain. Although MS remains the most common etiology for inflammatory demyelinating diseases of the central nervous system (CNS), the autoimmune disease neuromyelitis optica spectrum disorder (NMOSD) is a major differential diagnosis. The discovery of autoantibodies, such as aquaporin 4-IgG (AQP4-IgG) followed by myelin oligodendrocyte glycoprotein-IgG (MOG-IgG), and likely more to come [1,2], has further broadened the differential diagnosis of inflammatory demyelinating diseases. This is with the understanding that AQP4-antibody-associated NMOSD is frequently added to primarily inflammatory demyelinating diseases, although it is an astrocytopathy followed by oligodendrocytopathy and demyelination [3,4]. With more literature being published on MS and NMOSD, pattern recognition emerges. Pattern recognition not only affects the clinical manifestations of MS and NMOSD, such as recognizing the spectrum of optic neuritis, transverse myelitis, and brainstem syndrome, but also affects MRI findings in the brain, brainstem, spinal cord and the orbits. This review focuses on pattern recognition of these clinical presentations therefore our descriptive designation as the MS syndrome.

## 2. Brief Historical Overview of Multiple Sclerosis (MS) Diagnosis

The first diagnostic criteria for MS were introduced by Allison and Millar in 1954, followed by McAlpine in 1965. That same year, the Schumacher Committee formally published the first MS diagnostic criteria, heralding a half-century of intense research in the field of MS diagnosis, prognosis, pathophysiology, immunopathology, and treatment [5,6]. Due to the absence of a gold standard for unequivocally diagnosing MS, such as blood or cerebrospinal fluid (CSF) tests, the patterns of dissemination in time (DIT) (i.e., progression in time for primary progressive disease) and dissemination in space (DIS) have been considered diagnostic of the disease. These patterns at first relied on clinical data, limited paraclinical criteria [5,7], and subsequently on MRI [8,9,10,11]. Since the publication of the first McDonald criteria in 2001 [8], these diagnostic criteria have undergone numerous modifications but the criteria of DIS and DIT by clinical and/or MRI remain paramount to the diagnosis (Supplementary Material Table S1). Today, MRI of the brain and spinal cord is used to diagnose and prognosticate MS pre- and post-treatment. The emergence of disease-modifying therapies, with proven effectiveness in clinically isolated syndrome and MS, has called for further refinement of MRI criteria with exceptional sensitivity, specificity, and accuracy, thus allowing for an earlier diagnosis of the disease. Nevertheless, confusion of other inflammatory demyelinating diseases with MS remains problematic, particularly with practitioners who do not commonly see demyelinating diseases.

## 3. Overview of Neuromyelitis Optica Spectrum Disorder

The presence of a longitudinally extensive transverse myelitis (LETM) typically alerts the neurologist to the diagnosis of NMOSD, which is confirmed by testing positive for the neuromyelitis optica or the APQ4 antibody [12]. However, short segment spinal cord lesions (SSSCLs), that might not be unusual in early [13] and seronegative NMOSD can be easily confused with MS. Clinical presentation with bilateral simultaneous or sequential optic neuritis, with or without transverse myelitis, is highly suggestive of NMOSD. However, longitudinally extensive optic neuritis (LEON) might be overlooked because of the lack of routine use of MRI for the orbits in the diagnosis of optic neuritis. The differential diagnosis of a large edematous corpus callosal lesion is broad, and includes lymphomas, tumors, trauma, infections, metabolic (Marchiafava-Bignami) and vascular abnormalities, to cite a few [14], but the pattern is increasingly recognized in NMOSD (Table S2 and Figure S3a,b) [15,16]. Area postrema syndrome (Figure S4a,b), another core clinical presentation of NMOSD can be easily mistaken for a gastrointestinal illness in the hands of non-neurologists. Because of the pleomorphic presentation of demyelination and its variable outcome, there is a lack of unanimity between MS/NMOSD experts. A study by Jurynczyk et al. evaluated the agreement between different MS and NMOSD experts on the diagnosis of the seronegative AQP4-antibody NMOSD, MS and overlapping syndrome. Not surprisingly, the mean proportion of agreement for the diagnosis was low (ρ_0_ = 0.51) and ranged from 0.25 to 0.73 for individual patients. Clinical presentations associated with very low agreement (ρ_0_ < 0.5) included optic neuritis with limited recovery and short transverse myelitis, mild optic neuritis with short transverse myelitis and normal brain MRI, optic neuritis and borderline LETM, optic neuritis and transverse myelitis with brain lesions not fully typical of MS or NMO, and monophasic acute disseminated encephalomyelitis (ADEM)-like with optic neuritis and LETM [17].

**Figure 1:**
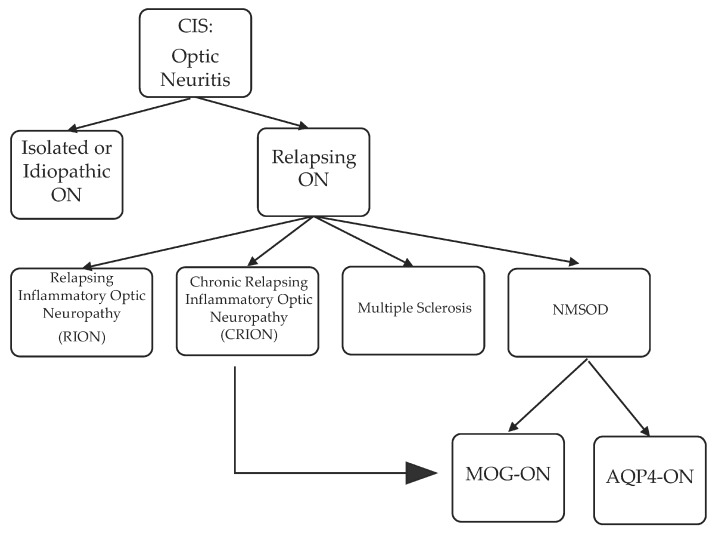
Evolution of clinically isolated syndrome (CIS) optic neuritis: The majority of patients with ON will evolve into RION, CRION, MS or NMOSD. A percentage of patients will stay as isolated ON. Furthermore, there is more data that at least a subset of patients with CRION are indeed MOG-antibody associated NMOSD. AQP4-ON: Aquaporin 4-antibody-associated optic neuritis; MOG-ON: Myelin oligodendrocyte glycoprotein-antibody-associated optic neuritis.

**Figure 2:**
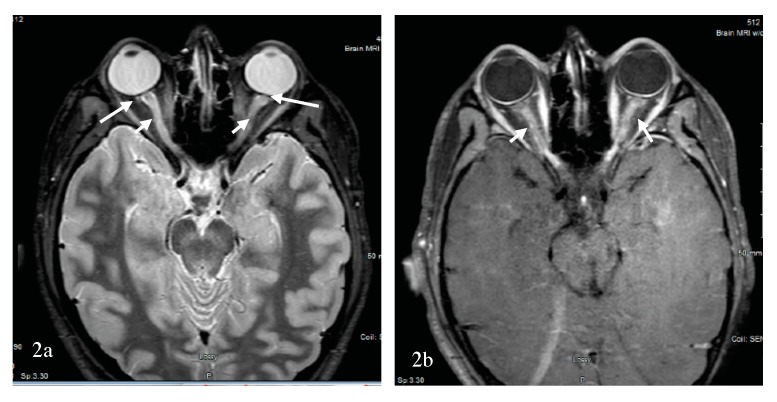
Axial short tau inversion recovery (STIR and T1 with contrast orbital MRI of an 18-year-old Caucasian male, with bilateral longitudinally extensive (small arrows) optic neuritis with anterior predominance, perineural sheath swelling (long arrows) and tilting and twisting of both optic nerves better seen on axial STIR (**2a,2b**). Serum MOG-antibody was positive.

**Figure 3:**
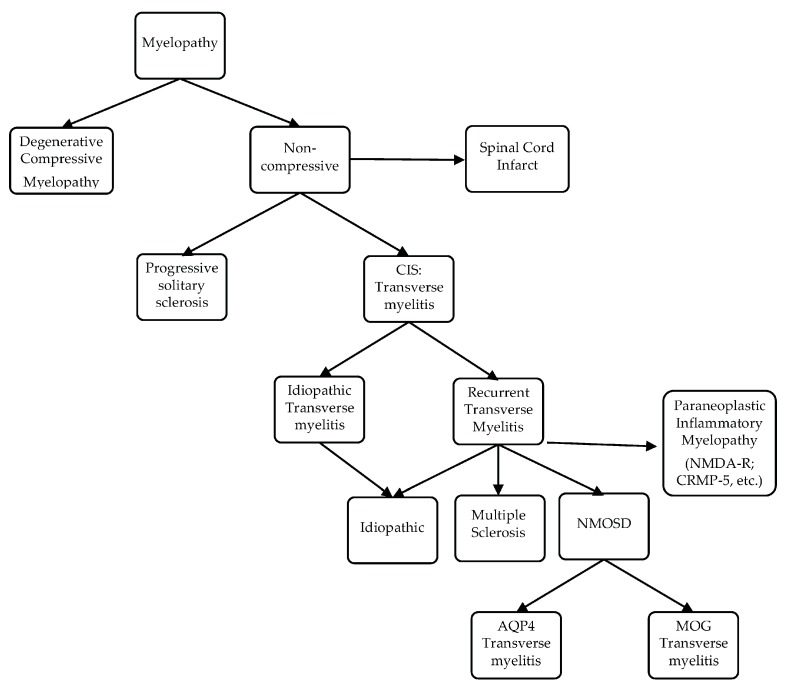
Evolution of clinically isolated syndrome (CIS) transverse myelitis.

**Figure 4:**
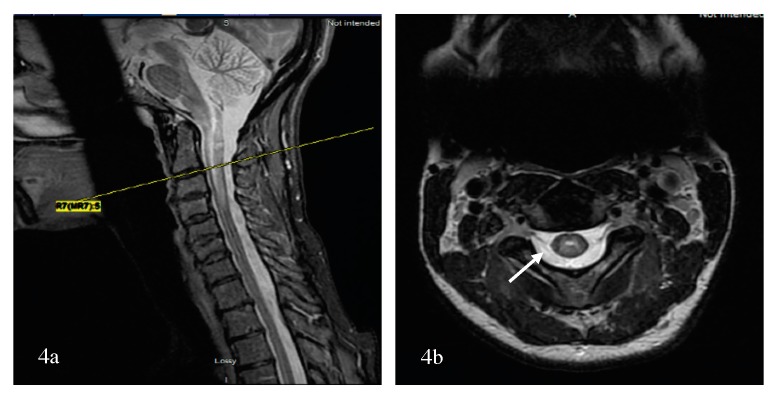
55-year-old African-American female, with AQP4-NMOSD; sagittal STIR cervical MRI demonstrates simultaneous linear lesion and LETM from the medulla to C4 (**4a**) seen at the center of the cord on axial T2 (**4b**).

Brain and spinal cord MRI have a significant role in differentiating MS from NMOSD, but there remains a group with demyelinating disease where the separation remains challenging. For instance, Barkhof’s criteria for DIS have been fulfilled by 5–42% of patients with NMOSD [18,19,20,21]. This MRI overlap between MS and NMOSD extends to both the AQP4 antibody and MOG antibody-associated NMOSD [22]. Clinical, imaging and differentiating patterns that suggest and support NMOSD are examined below. A historical overview, pathophysiology, and pathogenicity of two important biomarkers that can differentiate the two conditions are included. Additionally, brain MRI findings characteristic of NMOSD are summarized in Supplementary Material Table S2 [18,19,20,23,24,25,26,27,28,29], Figures S1–S5 and Figure 1, Figure 2, Figure 3 and Figure 4.

### 3.1. AQP4-Antibody Positive NMOSD: Pathophysiology and Pathogenicity of Aquaporin 4 Neuromyelitis Optica Spectrum Disorder (AQP4-NMOSD)

In 1999, Wingerchuk et al. described the clinical, MRI, and CSF features of 71 patients with NMO, with emphasis on the severity of the disease [30]. A B-cell-mediated pathology was suspected due to the association of NMO with autoantibodies and B-cell-mediated diseases. In 2004, Lennon et al. described a new antibody, NMO-IgG, localizing to the blood brain barrier (BBB) that was a specific serological biomarker of NMO and high-risk syndromes suggestive of NMO, such as LETM and recurring severe optic neuritis. A subsequent study by the same group demonstrated that the water channel aquaporin 4 (AQP4) was the substrate for the NMO-IgG [31]. To clarify, different types of aquaporins have been involved in water homeostasis in the brain and were associated with vasogenic and cytotoxic edema. APQs are comprised of highly conserved monomers or units that form homotetramers. Each unit, or monomer, has eight membrane-embedded domains, six transmembrane helices, and two short helical segments with a C- and N-terminus on the cytoplasmic side. These membrane-embedded domains surround a narrow aqueous pore [32]. On the extracellular side, there are three loops (i.e., A, C and E), and on the intracellular side, there are two loops (i.e., B and D). There is also a highly conserved asparagine-proline-alanine motif responsible for the selective orientation of the water transportation and an aromatic/arginine (AR/R) selectivity filter that prevents the entry of other molecules with water across the water channel [33]. An integral protein of the astrocytic plasma membrane [34], human AQP4 is expressed by astrocytes, and other AQP4-containing cells, by alternative splicing in the following two major isoforms: a long isoform called “M1”, and a short isoform called “M23”. In general, a highly homologous structure is characteristic of all members of the AQP family [35]. In the case of AQP4, however, M1-AQP4 and M23-AQP4 form heterotetramers that further aggregate in the cell plasma membrane in supramolecular crystalline assemblies called an orthogonal array of particles (OAPs). The size, shape, and composition of OAPs depend on the relative amounts of M1- vs. M23-AQP4, with larger particles formed at an increased M23:M1 ratio [36].

AQP4 is highly concentrated in the foot processes that make contact with micropapillary endothelia that form the BBB and in ependymal cells at brain cerebrospinal interfaces [34]. AQP4 is upregulated during astrocytosis and certain scar-forming pathologies but is considerably reduced in NMO. Other AQP4-expressing tissues include epithelial cells in kidneys, airways, gastrointestinal organs, and, at low levels, in musculoskeletal cells; thus, the most recent reported cases of acute myopathies involve AQP4 associated with NMOSD [37,38,39]. The AQP4 antibody binds to the extracellular surface of the AQP4 receptor in the three-dimensional form of the epitopes rather than their linear form, a pattern that is typical in human autoimmune disorders [34]. Despite its polyclonal production, the APQ4 antibody shows preferential binding and greater affinity to OAPs and thus to M23-AQP4. The binding of AQP4-IgG1 to AQP4 leads to complement-dependent cytotoxicity [36] and antibody-dependent cellular cytotoxicity [40]. Efficient complement-dependent cytotoxicity requires AQP4 assembly in OAPs, and therefore, this mechanism is minimal for M1-AQP4-expressing cells. Importantly, high concentrations of AQP4-IgG were reported not to inhibit AQP4 water permeability and not to lead to cellular internalization of AQP4 or AQP4-antibody binding [41]. In vivo consequences of AQP4 binding to the AQP4 antibody results in complement dependent cytotoxicity axonal injury followed by recruitment of granulocytes first and macrophages second, further disrupting the BBB [34]. Astrocyte loss and inflammation, with degranulation of neutrophils and eosinophils, and cytokine release culminate into secondary damage to the oligodendrocytes, with demyelination and neuronal/axonal loss. Thus, AQP4-NMOSD is not primarily a demyelinating disorder, but is nevertheless lumped under the MS syndrome due to clinical phenotypic similarities. In 2006, the NMO diagnostic criteria were updated to incorporate patients with NMO who had extra optico-spinal disease and NMO-IgG as a biomarker. Almost a decade later, newer NMOSD guidelines were published [42]. During that decade, numerous studies were published regarding (1) other clinical and inaugural manifestations of the disease, (2) best diagnostic techniques for the antibody, currently the approved technique is the cell-based essay, (3) pathogenicity of NMO IgG, and (4) brain, spinal cord, and orbit MRI findings of the disease, to cite a few. Attempts took place to find other suspected antibodies, resulting in the anti-myelin oligodendrocyte glycoprotein antibodies (MOG antibodies). It transpires that MOG antibody is not only associated with NMOSD (MOG-NMOSD), but also other inflammatory demyelinating disorders, such as pediatric acute disseminated encephalomyelitis (ADEM), pediatric multiphasic disseminated encephalomyelitis (MDEM), ADEM/MDEM-optic neuritis complex, benign unilateral cerebral cortical encephalitis with epilepsy and overlap syndrome or NMOSD-encephalitis complex that will be described later. The quest for further antibodies (such as AQP1, NMDA-R antibodies, etc.) associated with NMOSD and other inflammatory demyelinating disorders remains a work in progress and the future holds more antibodies to come [1,2].

### 3.2. Pathophysiology and Pathogenicity of Myelin Oligodendrocyte Glycoprotein (MOG) Antibodies

MOG is a mammalian glycoprotein exclusively expressed in the CNS. This glycoprotein is limited to the external surface of myelin and the plasma membranes of oligodendrocytes, with its highest antigen density in the outermost lamellae of myelin sheaths, thus making MOG accessible to autoantibodies [43]. MOG belongs to the Ig superfamily, with a single extracellular immunoglobulin variable (IgV) domain, a transmembrane domain, a cytoplasmic loop, a membrane-associated region, and a cytoplasmic tail. In humans, 15 different alternatively spliced MOG isoforms have been detected. These isoforms have been localized to the cell surface, in the endoplasmic reticulum, in the endocytic system, or found in secreted form. The secreted form could have important effects in triggering autoimmunity if released into the CSF and then drained into the bloodstream [44]. MOG antibodies isolated from animal models of MS target a denatured MOG protein. Similar autoantibodies (both IgG and IgM) for denatured proteins were present in MS patients with low titers and did not correlate with disease activity [45,46,47]. Importantly, the presence of cell-based assays has allowed for the isolation and quantification of MOG antibodies against the native or conformational epitope of MOG (nMOG), located on the extracellular domain of the protein [48]. Anti-MOG antibodies are likely relevant to the pathophysiology of MS, considering that they are present in early states of the disease and are not an epiphenomenon. Anti-MOG antibodies also persist during the disease course and are likely relevant to long-term pathophysiology. Brilot et al. investigated the occurrence and biological activity of IgG and IgM autoantibodies against nMOG in the serum and CSF of 47 children (mean age 7.63 years) during their first acute demyelinating syndrome (19 with ADEM and 28 with clinically isolated syndrome). The serum and CSF of these children were taken at the same time and prior to any treatment. Control groups included healthy children, children with other neurological diseases, children with type I diabetes mellitus, and adult MS patients. Native MOG antibodies were present in 47% of children with a demyelinating event (ADEM or clinically isolated syndrome), 6.9% of children with other neurological diseases, and absent in healthy controls as well as adults with MS and children with type I diabetes mellitus. The presence of MOG antibodies in pediatric demyelination and other neurological diseases and their absence in type I diabetes, highlights that these antibodies are markers of demyelination and not immune dysregulation. Native MOG antibodies were produced peripherally and in the CNS. The serum and CSF was simultaneously analyzed for a cohort of eight children (five with clinically isolated syndrome and three with ADEM). All patients, except three with clinically isolated syndrome, showed reduced antibody titers in the CSF. Native MOG antibodies were cytotoxic in demyelinating patients using an in vitro antibody-dependent cellular cytotoxicity assay. Furthermore, native MOG antibody titers inversely correlated with age (*r* = −0.46), suggesting a temporal evolution of the MOG antibody [49]. This possible temporal evolution was subsequently studied in 78 pediatric cases of CNS disease (27 with ADEM, 18 with clinically isolated syndrome, 18 with relapsing-remitting MS, and 15 with other general neurological diseases), 188 adult cases (71 with MS, 43 with other general neurological diseases, 20 with clinically isolated syndrome, and 7 with ADEM), and 43 healthy controls. Increased MOG antibodies serum titers were observed in pediatric ADEM, and these increased titers were associated with a younger age of onset. Recovery from ADEM was associated with a decrease in MOG antibody titers at last follow-up, with seroreversion in one patient. Incomplete recovery from ADEM was associated with a persistently increased antibody titer and reduced fluctuations in titer levels. Seroreversion was observed in patients with clinically isolated syndrome. Longitudinal analysis of nine patients with MS revealed stably low titers; three nevertheless became seropositive with time, with persistently low titers, which indicated ongoing CNS inflammation. Antibodies against nMOG were present in the CSF when serum titers were high, suggesting a peripheral production of MOG antibodies [50]. The persistence of MOG antibodies during remission suggests that, in isolation, these antibodies may be insufficient for disease activity [51]. Lately, MOG-antibody associated ADEM was reported in 2 adults. This further highlights the fact that the clinical spectrum of MOG-antibody associated diseases remains to be defined [52].

## 4. Optic Neuritis: From Clinically Isolated Syndrome to MS, NMOSD and Others

Although MS remains the most common etiology of ON, other etiologies are possible and summarized in Figure 1.

### 4.1. Single Inflammatory Optic Neuritis (SION), Relapsing Inflammatory Optic Neuritis (RION), and Chronic Relapsing Inflammatory Optic Neuropathy (CRION): “Formes frustes” of MS or NMOSD?

The term idiopathic [53] or isolated optic neuritis is somewhat loosely used in the literature to refer to optic neuritis without evidence of MS, NMOSD, or other diseases. The diagnosis of idiopathic or isolated optic neuritis cannot be made with certainty when a patient first presents with ON. Idiopathic/isolated optic neuritis is frequently limited to one episode [54] and referred to in the literature as single inflammatory/isolated optic neuritis (SION) or monophasic isolated optic neuritis [55]. However, isolated optic neuritis might recur outside the context of MS and NMOSD; while consensus regarding their presence is lacking, the following two forms of relapsing optic neuritis have been reported in the literature: relapsing inflammatory or isolated optic neuritis (RION) [55] and chronic relapsing inflammatory optic neuropathy (CRION). The existence of RION is debatable, considering that the conversion to MS, NMOSD, or other diseases may only be a matter of time. However, more studies on RION are being published. For example, analyzing the clinical and demographic criteria for 62 patients with RION, Benoilid et al. found that 40 patients (64.5%) did not convert to MS, NMO, or other autoimmune diseases over eight years of follow-up [56]. Furthermore, the natural history of RION was studied in 72 patients with two or more episodes of optic neuritis. Specifically, in a study by Pirko et al., the one-, five- and 10-year conversion rate of optic neuritis to MS was 2.8%, 14.4%, and 29.8%, respectively, and the conversion rate of optic neuritis to NMO was 5.6%, 12.5%, and 12.5%, respectively [57]. Predictors of RION converting to NMO included decreased visual acuity, shorter time to second relapse, more frequent relapses, and a significant female predilection. This study published in 2004 did not differentiate between AQP4- or MOG-antibody associated NMOSD.

CRION is differentiated from RION by the presence of progressive visual loss in between relapses and corticosteroid dependence, albeit there is no consensus regarding this latter criterion [56]. In 2003, the first case series of eight patients with CRION was published [58]. Subsequent reports in the literature were compiled in a systematic review of the clinical, laboratory, and imaging characteristics of 122 patients with CRION [59]. The age range for CRION is wide, spanning teenage to elderly years. Unilateral or bilateral, and simultaneous or sequential vision loss has also been reported with fellow eye involvement occurring days to decades later. The relapse rate for CRION is highly variable. Like all optic neuritis, pain and/or headache herald the condition and can be sleep disruptive. Visual loss at onset is variable from none to complete. The optic disc may be normal, swollen, or atrophic. Findings on visual field testing are variable, similar to MS-ON. Interestingly, uveitis has been reported conjointly in approximately 7% of cases [60]. Therefore, extensive blood testing should be performed to rule out systemic diseases. Notably, CSF analysis is typically normal. MRI of the orbits findings vary from normal to the presence swelling at the optic nerve head and contrast enhancement, T2 hyperintensity, and/or optic atrophy. Brain MRI is typically normal [59]. Recently, a number of patients with CRION or RION were found to have the MOG antibody [43,51,61,62]. In summary, the most notable distinguishing characteristics between RION and CRION available to date remains the progression in between relapses and steroid responsiveness, recognizing the lack of agreement on the definition. Furthermore, isolated monophasic and recurrent optic neuritis seem to exist as stand-alone entities. Lastly, time will tell whether all CRION cases are MOG-antibody associated optic neuritis or not.

### 4.2. Multiple Sclerosis-Associated Optic Neuritis (MS-ON)

MS-ON is a common presentation of MS in approximately 20% of patients [63]. The lifetime prevalence of MS-ON is 50–66% [64,65,66,67]. Clinical presentation of retro-orbital, peri-orbital, or oculomotor pain followed by subacute and varying degrees of visual loss in a young person are hallmarks of optic neuritis. Confounding factors leading to delayed diagnosis include minimal to no visual loss with decreased color sensitivity only on examination, the absence of pain, and the presence of positive visual symptoms in a person with or without a prior history of migraines. While painful visual loss appears to be the hallmark of optic neuritis, pain has also been reported in 12% of patients with anterior ischemic optic neuropathy [68]. Further, much information on inflammatory optic neuropathy results from the optic neuritis treatment trial [69]. In a 1992 study by Beck et al., 457 patients were randomly assigned to placebo, oral prednisone at a dose of 1 mg/kg/d for two weeks or high-dose intravenous methylprednisolone (IVMP) for three days followed by an oral taper. The patients were monitored for six months; 77.2% of the subjects were women. Pain, which was present in more than 92% of the cases, preceded the visual symptoms and was unrelated to the presence or absence of optic disc swelling. Visual acuity loss was almost equally distributed between mild (≤20/40), moderate (20/50–20/190), and severe (≥20/200). Complete visual loss was present in 10% of patients. Noticeably, even when visual acuity was 20/20 or better, many patients had other abnormalities, such as decreased contrast sensitivity and/or abnormal color vision or unusual visual field tests. Fellow eye involvement was observed with decreased visual acuity and contrast sensitivity in 14% and 15% of patients, respectively, and abnormal color vision and visual defects were observed in 20% and 48% of patients, respectively. Disc swelling was noted in 25–40% of patients, depending on the time of the exam following the onset of symptoms, <5 days or ≥5 days. Positive visual symptoms, such as photopsias, were observed in 30% of patients. Notably, the pattern of painful visual loss and photopsias can be easily confused with migraines. The prolonged duration of visual loss and photopsias should alert the clinician to an alternative diagnosis considering that it is uncommon for a migraine aura to last for days and persists beyond the pain [70]. Visual field cut in patients with optic neuritis was not only centrocecal and central, but also paracentral, altitudinal, quadrantic, hemianopic, peripheral, arcuate or double arcuate, enlarged blind spot, nasal, and vertical step. Visual recovery, including acuity, contrast sensitivity, color vision and field-testing, was faster in the IVMP group. At six months, visual acuity was the same in the three groups, but the difference in low-contrast sensitivity, color vision, and field-testing persisted between the IVMP and the other groups. Only 5–7% of the patients in all groups had visual acuity of 20/50 or worse. Furthermore, only one out of 457 cases had a compressive optic neuropathy due to a pituitary tumor diagnosed by MRI. Based on these findings, the authors of the optic neuritis treatment trial did not recommend an MRI of the brain to diagnose optic neuritis unless an atypical course alerts the clinician for further imaging study [71].

### 4.3. Neuromyelitis Optica Spectrum Disorder-Associated Optic Neuritis

Optic neuritis is a presenting sign of NMOSD more than 50% of the time. However, the difference between MS-ON and NMOSD-ON might not be evident with the first attack of optic neuritis if the brain MRI is negative. Fortunately, an orbital MRI performed early and prior to any treatment can facilitate diagnosis and is commonly abnormal in NMOSD-ON cases [53]. Orbital MRI, however, was not performed in the optic neuritis treatment trial [72], although the technology was available [73]. A 2016 study found that the MRI of the orbits was less likely to be performed in MS-suspected optic neuritis when a neurologist, rather than an ophthalmologist, saw the patient [74]. MRI of the spinal cord might foretell the diagnosis by further disseminating the patient in space (i.e., DIS). However, if no orbital MRI is completed, and there are no further signs of DIS, the diagnosis is obscured a priori. It has been suggested that antibody testing for AQP4-IgG be reserved to patients with severe visual loss, poor visual recovery, bilateral or sequential visual involvement, recurrent optic neuritis [75] and unique findings on the MRI of the orbits. The efficiency of MRI of the brain and anterior visual pathways for differentiating NMOSD-ON from MS-ON was retrospectively examined in a study [76]. Brain MRI was included to examine the ability of DIS per the 2010 McDonald criteria [9] to differentiate between the two conditions. The absence of brain DIS, longer optic nerve lesions, an increased number of segments involved, and optic chiasma and tracts involvement were suggestive of NMOSD-ON. Further, Buch et al. examined the sensitivity and specificity for a combination of these criteria. Specifically, bilateral optic nerve, chiasma or optic tracts, and three or more segment involvement in the absence of MS-like lesions with DIS were suggestive of NMOSD-ON, with a sensitivity of 69% and a specificity of 97% [76]. More specifically, a longitudinally extensive optic neuritis (LEON) with chiasma and optic tract involvement was found to be strongly suggestive of AQP4-ON [77]. Not all LEON, however, are associated with AQP4 antibody. The test sensitivity of AQP4 antibody in the serum is about 50–80%, and MOG antibodies have been described in 25% of cases of AQP4 antibody seronegative NMOSD and cases of CRION [55]. In a study of optic neuritis by Akaishi et al., the cross-sectional prevalence of AQP4-ON, MOG-ON, MS-ON, and RION was 30–35%, 25–30%, 25–30%, and 10–15%, respectively [74]. Here, Akaishi et al. reported a comparative study of the clinical, laboratory, and imaging findings of MS-ON, AQP4-ON, MOG-ON, and RION. Female dominance was overwhelming in the AQP4 group, at 98% vs 80% in the MS group and 50% in the MOG group, which is different from other reports [43,51]. While the mean age of onset of MS-ON was less than 50 years for the entire cohort, patients with AQP4-ON had greater mean age of onset. A broader age distribution existed in the MOG-ON group, with optic neuritis diagnosed in children and the elderly similar to MOG-NMOSD. The number of optic nerve segments involved during the acute phase of optic neuritis in all groups was assessed. To clarify, the optic nerve has been divided into the following six segments anteriorly to posteriorly: (1) pre-orbital, (2) retro-orbital, (3) canalicular, (4) intracranial, (5) chiasmatic, and (6) retrochiasmatic or optic tract portion [74,77]. On MRI of the orbits, MOG-ON showed longitudinally extensive contrast enhancement, with severe swelling and a twisted running. The inflammation was anterior, with 70–80% intraorbital perineurial contrast enhancement. An example of MOG-ON is shown in Figure 2.

AQP4-ON was longitudinally extensive, with greater posterior involvement including the canalicular, chiasmatic, and retrochiasmatic segments, but with milder swelling and rare twisting. The MS-ON contrast enhancement was less extensive, with a median of only a couple of segments involved [74], as confirmed by others [77]. Optic nerve head swelling has been observed clinically with MOG-ON [78]. Significant decrease in visual acuity was associated with AQP4-ON followed by MOG-ON and MS-ON. In both the MOG-ON and the MS-ON groups, visual acuity loss during a relapse and the long-term outcome past one year were similar, but they were worse in AQP4-ON group. The less severe prognosis of MOG-ON was confirmed in a study by Matsuda et al., who also showed that the residual deficit was commonly present due to an increased number of relapses per year [62]. A 2017 study by Stiebel-Kalish et al. compared the visual acuity, field defect, and thickness of the retinal nerve fiber layer over time between a group of MOG-ON and AQP4-ON. In the MOG-ON group, the final visual acuity, mean visual field defect, and retinal nerve fiber layers were preserved, while adjusting for the number of relapses [79]. In a separate study by Havla et al., the optical coherence tomography analysis of eight patients with MOG-ON demonstrated a reduced papillary retinal nerve fiber layer compared to MS-ON; this study also revealed the presence of microcytic macular edema in six patients with MOG-ON and in two patients with AQP4-ON. Fellow eye was also affected in MOG-ON [80]. The favorable long-term prognosis of MOG-ON was not replicated in one of the largest cohort of patients (*n* = 50) with MOG-NMOSD [81]. Although short-term visual acuity was improved in patients with MOG-ON, this long-term outcome was not confirmed compared to AQP4-ON, a discrepancy that was explained by the increased number of relapses and the lack of corticosteroid use in some of these patients with MOG-ON. Regardless, a study by Piccolo et al. found severe visual acuity loss at onset and at last follow-up in five-eighths of patients with MOG-ON [55]. Recently, worse vision-related quality of life in both AQP4- and MOG-NMOSD than in MS patients was reported, steered by patients with bilateral and severe ON in the NMOSD group. Additionally, OCT, visual function and vision-related QOL parameters were similar in AQP4- and MOG-NMOSD groups [82]. Overall, there is convergence of data that visual outcome from MOG-ON is not as favorable as MS-ON, but nevertheless the visual outcome from MOG-ON is more favorable than AQP4-ON. Similar findings were recently reported in 12 Chinese Han patients [83]. The exquisite steroid sensitivity of MOG-ON, reminiscent of that observed with CRION, raises the possibility that CRION could be a manifestation of the MOG-inflammatory demyelinating disease spectrum. This observation was indeed confirmed in more than one study where patients with a clinical diagnosis of CRION or RION were found to have the MOG antibody [43,51,61,62]. The potential course of demyelinating optic neuropathy is summarized in Figure 1. Additionally, a summary of pattern recognition of ON in MS and AQP4- and MOG-NMOSD on orbital MRI is provided in Table 1.

## 5. Transverse Myelitis Pattern Recognition: From Clinically Isolated Syndrome to MS, NMOSD and Others

### 5.1. Multiple Sclerosis-Associated Transverse Myelitis (MS-TM) and Myelopathy

#### 5.1.1. Acute Complete Transverse Myelitis (ACTM) versus Acute Partial Transverse Myelitis (APTM)

The distinction between complete and partial/incomplete transverse myelitis was highlighted a quarter of a century ago [84]. Acute *partial* transverse myelitis is characterized by an asymmetric or mild loss of function, which is in contrast to the involvement of all modalities in acute *complete* transverse myelitis and severe neurologic deficit. In 2002, the ATM Working Group proposed a series of laboratory tests to try to differentiate idiopathic from post-infectious or inflammatory transverse myelitis [85]. Although this list is not fully comprehensive, it provides a framework for the workup of ATM, and can represent a work in progress that will require occasional refinement to include new knowledge. Spine MRI findings differ between ACTM and APTM. Lesions in the former involve the whole cross section of the spinal cord or at least its center. Lesions in the latter are dorsolateral. The potential for APTM to evolve into MS was recognized early. Lesions longer than three vertebrae in length exist in MS but typically affect the dorsolateral tracts, an important differentiating factor from the longitudinally extensive transverse myelitis associated with NMOSD. While an abnormal MRI of the brain predicts the future conversion into MS [86,87,88], an estimated 20–30% of patients with APTM and a negative cerebral MRI will convert into MS [89,90]. The presence of oligoclonal bands in the CSF appears to increase the conversion likelihood [89].

#### 5.1.2. The Case of Progressive Solitary Sclerosis

A rare phenotype of demyelination reminiscent of primary progressive MS, i.e., progressive solitary sclerosis [91], consists of a solitary demyelinating CNS lesion most commonly located within the cervical spinal cord or cervico-medullary junction. Less commonly affected areas include the thoracic spinal cord, subcortical white matter, and ponto-mesencephalic junction. This spinal cord lesion is typically less than three vertebrae segments in length. Bilateral lesions involving the medullary pyramids or cervicomedullary junction present with quadriparesis but no brainstem symptomatology [92]. Cerebrospinal fluid analysis characteristics of MS are present in 50% of cases. Originally described in 7 patients [91], similar clinical, CSF and imaging findings were reported in 10 more patients [93,94,95,96]. Time will tell whether solitary sclerosis should belong to the MS disease or not.

### 5.2. Neuromyelitis Optica Spectrum Disorder-Associated Transverse Myelitis (NMOSD-TM) and Longitudinally Extensive Transverse Myelitis (LETM)

#### 5.2.1. LETM versus Spinal Cord Infarct versus Spondylotic Myelopathy

In up to 40% of cases, transverse myelitis can be the presenting manifestation of NMOSD [97,98] The presence of a LETM almost always evokes the diagnosis of AQP4-NMOSD. However, LETM has been associated with other inflammatory diseases of the CNS such as ADEM, MS, overlap syndromes (e.g., Sjogren’s and NMO), sarcoidosis, antiphospholipid syndrome, vasculitis [99], Behcet’s disease, and paraneoplastic syndrome, in addition to non-inflammatory etiologies such as intramedullary tumors, dural arteriovenous fistula, Alexander’s disease, metabolic and compressive myelopathies and spinal cord infarction [100,101]. An extraordinary challenge in the differential diagnosis of LETM is spinal cord infarction. A study by Kister et al. analyzed the clinical, demographic, and MRI characteristics of 11 cases with spinal cord infarction and 13 cases with LETM. More commonly associated with LETM were the female gender, non-White ethnicity, bright spotty lesions on MRI of the spinal cord described below, location within 7 cm of the cervicomedullary or cervicothoracic junction, extension to the pial surface, and contrast enhancement. Interestingly, patient age, lesion length and cross-sectional area, and cord expansion did not differentiate the two conditions [102]. Another challenging diagnosis is spondylotic myelopathy that might be confused or sometimes associated with myelitis. Flanagan et al. compiled the findings of 56 patients with the condition. A peculiar pattern of “transverse pancake gadolinium enhancement” is described caudal to the site of maximal stenosis and at the craniocaudal midpoint of a spindle-shaped T2 hyperintense lesion. On axial cuts, a complete or incomplete circumferential pattern of enhancement with gray matter sparing is observed. Distinctively, spondylotic myelopathy is associated with a prolonged contrast enhancement resolution that might extend for a year, post-surgical decompression [103].

The following sections focuse on factors differentiating idiopathic LETM, AQP4- and MOG-antibodies-associated LETM.

#### 5.2.2. Seropositive Versus Seronegative LETM: Does Seronegative LETM Truly Exist?

The answer to this question is a matter of debate in the literature. In an effort to define truly idiopathic and AQP4-antibody-associated LETM, a 2015 study by Hyun et al. enrolled 108 patients with first-ever LETM (mean follow-up periods between seropositive and negative groups were 5.4 ± 2.6 years vs. 7.0 ± 4.4 years). To determine the true seropositive and seronegative statuses, the AQP4 antibody status was repetitively confirmed by three different validated methodologies, discussed below [104]. CSF glial fibrillary acid protein (GFAP) levels were measured to investigate astrocytic damage. Of the 108 patients, 55 were positive for AQP4 antibodies (i.e., P-LETM) and 53 were consistently negative (i.e., N-LETM). Seven out of 53 N-LETM were later diagnosed with seronegative NMO (49%), and four were positive for MOG antibodies (8.2%). The remaining 42 patients (N-LETM) showed several features distinct from P-LETM, including male predominance, older age of onset, milder clinical presentation with partial transverse myelitis features, less frequent relapses, spinal cord confinement with shorter segments, and the absence of combined autoimmunity. While CSF GFAP levels were markedly elevated in P-LETM, they were not increased in N-LETM. In the group of N-LETM, 39% were true seronegative or idiopathic [104], consistent with an Italian study, which reported 41% idiopathic N-LETM among 37 first-ever LETM [105]. Interestingly, idiopathic LETM was not necessarily monophasic, although relapse rate was less than P-LETM [104]. Fewer patients were treated with immunosuppressants, most likely due to the misconception that idiopathic transverse myelitis is monophasic. The increased frequency of recurrent N-LETM in the study by Hyun et al. compared to previous studies may be due to the longer duration of follow-up. Disease heterogeneity was noted in the N-LETM group where severe cases were present [104]. A study by Kitley et al. compared P-LETM and N-LETM, and produced discrepant results. In this cohort of 76 patients presenting with LETM, 58% (*n* = 44) had the AQP4 antibody and 42% (*n* = 32) were negative. The two groups were followed for a median of 61.35 months (with a range of 2.3–260.2 months) and 25.04 months (with a range of 1.9–169.4 months), for AQP4-antibody-positive and AQP4-antibody-negative respectively. In this series, however, most of the AQP4-antibody-negative group had an identifiable etiology unlike the above two series. Six of 32 had the MOG antibody (18.75%), five had ADEM, and the rest had vasculitis, leptomeningeal syndrome, infections, paraneoplastic disease, and spinal cord infarction. The final rate of true idiopathic N-LETM was 6.5% and true N-LETM could not be clinically differentiated from P-LETM [106].

Albeit less common than AQP4-LETM, MOG-LETM is turning out to be an important differential diagnosis of N-LETM, and clinical features are crucial in differentiating these two conditions. However, the MOG antibody assay is not available commercially, and the prevalence of MOG-LETM is variable depending on the series studied. In a 2016 study by Cobo-Galvo et al., 13 cases of MOG-LETM were compared to 43 cases of N-LETM [107]. Distinctive clinical features in the MOG-seropositive group included the following: younger age at onset, increased predisposition to optic neuritis relapses, and improved prognosis. A total of 23% of patients who presented with a first episode of N-LETM tested positive for the MOG antibody [107] vs. 18.75% in another study [106].These frequencies, which are greater than previously described (8.2%; [104]), may be explained by discrepancies in the definition of LETM, which was undefined in the Hyun et al. study, as well as a genetic predisposition and unintentional selection bias. The Cobo-Calvo study had younger N-LETM patients with a more homogeneous ethnic background and followed an acknowledged definition for LETM (≥three vertebral segments). A large and comprehensive workup was also performed to rule out alternative diagnoses. Equal involvement of male gender and steroid sensitivity were noted similar to that observed with MOG-ON [74]. The clinical course of MOG-LETM patients was less severe compared to AQP4-antibody seropositive or truly seronegative forms of NMOSD, despite the similar frequency of severe episodes at onset and the increased relapse rate during the follow-up. Similar to AQP4-LETM cases, a spinal cord lesion evanescence by MRI was observed in a significant proportion of cases. A possible explanation for this recovery is the effect of the MOG antibody itself. Indeed, the intracerebral injection of the human MOG antibody in mice causes few and transient myelin changes, alteration of axonal protein expression without leukocyte infiltration, and recovery within two weeks [107]. The German Study, described in detail later, reported the findings on spinal cord MRI in MOG-NMOSD [22]. The median length of the LETM and the short segments transverse myelitis (SSTM) was 4 and 1.5 vertebral segments, respectively. Swelling and contrast enhancement were commonly present in 70.4% and 67.9% of transverse myelitis cases, respectively. The cervical spinal cord was most commonly affected, followed by the thoracolumbar areas. Other reports, however, emphasized the thoracolumbar involvement [108], particularly the conus [109]. Cord lesions were equally distributed centrally and peripherally. Asymptomatic spinal cord lesions were also present. In summary, the clinical and MRI phenotypes of MOG-LETM are reminiscent of AQP4-LETM, but a male prevalence and a less aggressive course are differentiating factors. Additionally, it appears that MOG-LETM accounts for about 20% of previously reported seronegative LETM. Furthermore, the existence of idiopathic LETM remains arguable. Further refining its definition and the serological assays, longer follow-ups, and the identification of more antibodies will likely decrease this group. In addition to its dubious nature, there needs to be a close follow-up of patients affected by N-LETM to determine long-term management, considering that the diagnosis of idiopathic LETM is associated with less potential for long-term treatment.

#### 5.2.3. Short Segment Transverse Myelitis (SSTM) in Neuromyelitis Optica Spectrum Disorder

The presence of a short segment (<3 vertebral segments) transverse myelitis (i.e., SSTM) was reported in 14% of patients with AQP4-NMOSD [110] and most recently in patients with MOG-NMOSD [22,111]. SSTM can occur early in NMOSD, with immunosuppressive treatment and due to MRI timing [13]. Particularly, an early MRI might detect a lesion at its beginning, and a late MRI might capture the lesion following improvement. Not surprisingly, a delay in the diagnosis and treatment of the SSTM associated with NMOSD in comparison to LETM is common. However, 92% of the SSTMs were followed by a LETM. Aside from the presence of the AQP4 antibody, which is confirmatory for the SSTM disease, there are several clinical features that suggest its diagnosis, including the following: non-White ethnicity, advanced age, personal history of autoimmunity and tonic spasms, prior history of severe and bilateral optic neuritis with limited recovery, prior episode of uncontrollable nausea and vomiting, and lastly, the absence of oligoclonal bands in the CSF. Additionally, radiological features that suggest the diagnosis of SSTM include a central lesion associated with T1 hypointensity and the absence of typical MS brain lesions [110]. Interestingly, the frequency of SSTM at initial presentation was recently reported in 14.5% of 76 patients subsequently diagnosed with AQP4-NMOSD [112]. Thus, SSTM is not a rare event in NMOSD, and clinical, paraclinical, and imaging features suggestive of NMOSD are key to the diagnosis.

#### 5.2.4. Imaging Patterns of Neuromyelitis Optica Spectrum Disorder-Associated Transverse Myelitis (NMOSD-TM)

##### Linear Lesions in NMOSD

Linear lesions are defined as limited ependymal inflammation in the medulla, which is due to weakness of the fluid-BBB, spinal cord, or both. While studying the relationship between linear lesions and LETM, most patients with NMOSD show linear lesions preceding LETM [113]. This raises the possibility that linear lesions are precursors to LETM. Further, the simultaneous presence of linear lesions and LETM, or linear lesions following LETM, might reflect a more severe degree of inflammation [113].

##### Bright Spotty Lesions (BSLs) in Neuromyelitis Optica Spectrum Disorder

**Figure 5:**
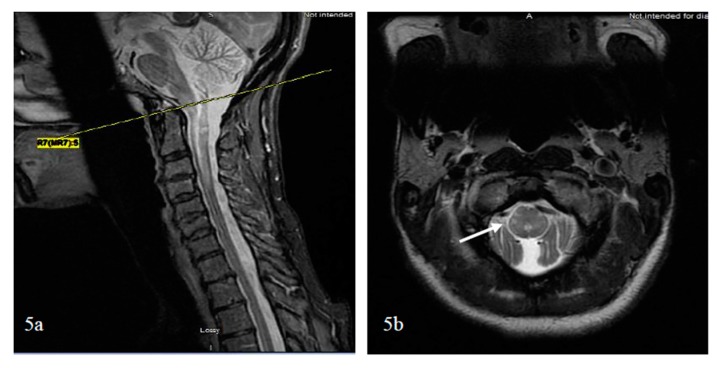
Added to above paragraph 55-year-old African-American female, with AQP4-NMOSD sagittal STIR cervical MRI demonstrates simultaneous linear lesion and LETM from the medulla to C4 (**5a**). An axial cut at the level of the cervicomedullary junction, axial T2 demonstrates 4 peripheral bright spotty lesions (**5b**).

Bright spotty lesions (BSLs) on the spinal cord were observed with or without LETM and in acute and chronic disease states. BSLs were found in 54% of patients with NMSOD (*n* = 24 patients) and 3% patients with MS (*n* = 34) [114]. BSLs are best visualized on axial cuts with T1- and T2-weighted imaging, where they present as hypointense and hyperintense lesions, respectively, and are located centrally or peripherally (Figure 5). Their hypointensity on T1-weighted imaging, similar to or greater than the CSF, and their contrast enhancement might reflect a destructive damage predominantly to the gray matter and blood spinal cord barrier resulting in microcystic changes [114].

To further differentiate LETM (≥three vertebral segments) associated with NMOSD, MS, and other neurological diseases of the spinal cord, the most useful MRI characteristics, as found in a study by Pekcevik et al., were the presence of punctate or large cavities BSL, T1 “dark” lesions, and large lesions involving more than 50% of the spinal cord cross section [115]. In the two studies, the sensitivity of BSLs was 88% and 65%, and the specificity of BSLs was 97% and 89%, respectively [114,115]. There was an emphasis on T1-dark rather than T1-hypointense lesions due to inter-observer disagreement on the lesion definition for T1 hypointense. The presence of greater than 50% of cross-sectional involvement or “transversally” extensive lesions was sensitive, but had poor specificity. Other factors that could not differentiate between the three groups included the presence or absence and the pattern of contrast enhancement (well-defined homogeneous or ill-defined heterogeneous), as well as spinal cord abnormality extending into the brainstem [115]. In summary, BSLs are imaging markers of spinal cord lesions associated with NMOSD and can be present in isolation or in conjunction with LETM; again, their darkness on T1 should evoke the diagnosis considering that dark or severe hypointense lesions on T1 are rarely detected visually in transverse myelitis associated with MS [116]. The potential course of demyelinating transverse myelopathy is summarized in Figure 3. Additionally, a summary of pattern recognition of TM in MS and AQP4- and MOG-NMOSD on spinal cord MRI is provided in Table 2.

## 6. Brainstem and Cerebellar Pattern Recognition: From Clinically Isolated Syndrome to MS, NMOSD and Others

### 6.1. Multiple Sclerosis-Associated Brainstem and Cerebellar Symptoms

At onset, optic neuritis, transverse myelitis and vertigo, diplopia, and ataxia in a person between the ages of 20 and 50 years are quite suggestive of MS. However, although uncommon, isolated brainstem presentation can be misleading and includes oculomotor nerve palsy, trigeminal neuralgia, facial nerve palsy of the “peripheral type” and hemifacial spasms, which require a thorough workup to rule out life-threatening conditions such as an aneurysm or a brainstem tumor [118]. Asymptomatic brainstem involvement is not unusual either. Like optic neuritis and transverse myelitis, acute demyelinating brainstem syndrome can remain isolated or can evolve into MS, NMOSD, or recurrent brainstem encephalitis [119]. On imaging, acute posterior fossa lesions in MS present with T2 hyperintensity and contrast enhancement. Chronic lesions might show continuous T2 hyperintensity, but it is not unusual for them to also show focal atrophy. The presence of focal lesions and atrophy, however, is unspecific and is seen in a number of pathologies [120].

#### 6.1.1. Trigeminal Neuralgia or Facial Sensory Loss

Trigeminal neuralgia at onset of MS is rare, and accounts for <1% of initial MS presentations [121]. However, using routine MRI of the brain in patients with MS (pwMS), trigeminal nerve enhancement was reported in 24 of 851 (2.8%) patients, with bilaterality in two-thirds of the patients and extension into Meckel’s cavum in 19 patients [122]. The nerve enhancement was partial or complete, involving the nerve across its length, from its pontine exit zone (i.e., root entry zone myelinated by oligodendrocytes), passing by the central-peripheral transitional zone, and up to the Meckel’s cavum (i.e., myelinated by Schwann cells). This indicates that involvement of peripheral myelin occurs in MS in addition to the central portion in relation to the dorsal root entry zone, which is supported by pathological studies [122,123]. A different study conducted by da Silva et al. found a similar frequency of trigeminal nerve enhancement (2.9% of a cohort of 275 MS patients) with bilateral involvement (75% of the cohort) [124]. Trigeminal nerve enhancement visualized by MRI is occasionally associated with sensory symptoms of pain, anesthesia, or paresthesias [124]. A third study by Mills et al. reported an increased prevalence of trigeminal nerve involvement in 11 of 47 patients (23%) using 3T MRI of the brain, with 1 mm slices through the posterior fossa [123]. Specifically, the intracranial trigeminal nerve pathway was mapped and showed T2 hyperintensity in the trigeminal root entry zone and intrapontine tract, with potential extension in either direction to the trans-cisternal portion of the nerve and what was thought to be the trigeminal nuclei (both ascending and descending). The changes were often bilateral (50% cases) and symmetrical. In this study, all the patients were asymptomatic [123]. In a study by Swinnen et al. involving 43 pwMS or clinically isolated syndrome referred for trigeminal nerve symptoms, the MRI of the brain demonstrated a linear plaque involving the intra-pontine fascicular portion of the nerve and lesions involving the spinal nucleus and tract in 48.8% and 53.4% of the patients, respectively. Lesions of the principal sensory nucleus and mesencephalic nucleus of the trigeminal nerve were less common (12–33%). In this study, however, lesions were most often unilateral (80% of the cases) [125]. In summary, uni- and bilateral trigeminal nerve enhancement is not unusually observed on the MRI of pwMS patients, albeit asymptomatic clinically, and is an example of central and peripheral myelin involvement in MS.

#### 6.1.2. Oculomotor Abnormalities

Unilateral or bilateral internuclear ophthalmoplegia, the most common oculomotor abnormality in MS and a hallmark of the disease in a person aged 20–50 years, incites practitioners to actively follow the patient even when brain imaging is normal. [118]. However, isolated sixth nerve palsy is very rare and was reported in three out 600 pwMS seen at a neuro-ophthalmology clinic. Usually a brainstem lesion that affects the sixth nerve nucleus results in additional deficits due to the intimate relationship of the fascicular fibers to other pontine structures [126]. Isolated fourth nerve palsy is also very rare. Besides the difficulty in diagnosing superior oblique palsy, the condition is rare because the fascicular course of the trochlear nerve is exposed to little myelin [127]. Other combinations of oculomotor abnormalities occur in MS both acutely and chronically, including as one-and-a-half syndrome and walled-eyes bilateral internuclear ophthalmoplegia (WEBINO). A 2016 report detailed a list of oculomotor abnormalities and ocular instabilities observed in pwMS, including a systematic approach to their diagnosis [128]. Periaqueductal lesions, commonly seen in NMOSD, were described in 19.4% of pwMS patients and were associated with oculomotor abnormalities and higher brainstem disability scores. Some of these lesions were wedge-shaped (42%), and others had an abnormally hyperintense broad peri-aqueductal gray rim; a third group had both characteristics, meaning severe involvement. Contrast enhancement was absent. Notably, a three-dimensional direct inversion recovery technique is optimal in allowing for a strong contrast between periaqueductal gray and surrounding tissue, due to a suppression of the CSF and white matter. The pathophysiology of these lesions in the periaqueductal area is likely to involve inflammation around the subependymal veins, similar to the areas around the lateral ventricles. Moreover, the close vicinity to the CSF and potential direct gliotoxic effects from the CSF might be an additional mechanism for the formation of periacqueductal lesion in MS [129].

#### 6.1.3. Peripheral Type Facial Nerve Palsy

The frequency of facial nerve palsy at the onset of MS varies from 1.4–4.8% [130]. In peripheral seventh nerve palsy, the lesion of the nerve usually occurs at the level of the geniculate ganglion (located in the facial canal) and therefore outside of the CNS. Peripheral facial palsy, however, can also result from a central lesion at the level of the ipsilateral facial nucleus or facial nerve at the pons [131].

#### 6.1.4. Cerebellar Symptoms

Clinically isolated cerebellar syndrome is rare in MS, but cerebellar involvement is very common in advanced disease states and in pathological studies, even when brain imaging antemortem does not show any cerebellar findings [132]. Brainstem lesions frequently affect the cerebellum with its afferent and efferent tracts [133]. The clinical manifestations of cerebellar pathology depend on the lesion site and include truncal and appendicular ataxia, eye movement abnormalities, cognitive impairment, and tremors, which are the most common symptom. Common lesion locations include middle cerebellar peduncles and cerebellar hemispheric white matter [120].

### 6.2. Acute Brainstem Syndrome Associated with Neuromyelitis Optica Spectrum Disorder

NMOSD-associated acute brainstem syndrome might be difficult to diagnose, particularly when the brainstem syndrome is a precursor to NMOSD. Notably, brainstem syndromes in NMOSD have a peculiar pattern that is likely easy to diagnose by a neurologist who is familiar with the presentation, but might represent a challenge in a gastroenterology clinic. In the latest clinical criteria for NMOSD [42], the importance of acute brainstem syndrome was highlighted by making it one of the core clinical criteria for diagnosing seropositive NMOSD; the dorsal medullary or area postrema syndrome was one of two very specific criteria required for diagnosing seronegative NMOSD [42].

#### 6.2.1. Intractable Hiccups, Nausea and Vomiting

In NMOSD, intractable hiccups and nausea preceded (54% of cases) or accompanied (29% of cases) neurological symptoms such as optic neuritis and transverse myelitis. Occasionally, a significant increase was reported in AQP4-IgG titers. Medullary involvement based on MRI, in addition to short or long segment spinal cord lesions, were present in about 50% of the cases [134]. Similarly, the initial symptom for 12 patients with NMOSD was intractable vomiting for three months prior to the onset of optic neuritis or transverse myelitis. The clinical and neuroimaging observations were consistent with area postrema involvement, a circumventricular organ that lacks the BBB thus allowing diffusion of stimulating IgG into the CNS [135]. Both intractable hiccups and vomiting were completely resolved with corticosteroids [134,135]. Heralding brainstem symptoms in demyelinating diseases are not uncommon. In a study by Cheng et al. involving 352 patients with CNS demyelinating diseases, 31 patients (8.8%) presented with an acute brainstem syndrome. The AQP4 antibody was present in only 14 of these 31 patients (45%). Intractable hiccups, nausea, and vomiting occurred more often in the positive group. Also in the positive group, five out of 14 patients had recurrent brainstem symptoms before optic neuritis or transverse myelitis vs. one out of 17 in the negative group. Dorsal medullary lesions were more often present in the positive rather than the negative group, but midbrain and pons were equally affected in the two groups. None of the 31 patients with acute brainstem syndrome had spinal cord lesions at onset, although LETM was commonly found in the positive group during follow-up. Over two years, 100% of the positive group and 17.65% of the negative group converted to NMOSD (i.e., 17 of 31 of the total group). Furthermore, seven of the 31 converted to MS, and the remaining 7 had no further neurological events. While the Expanded Disability Status Scale (EDSS) was similar at baseline, the positive group had increased EDSS at last follow-up, underlining the importance of AQP4-antibody testing for diagnosis and prognosis [136]. Not unexpectedly, in a cohort of Chinese patients with NMOSD, medullary involvement was associated with an increased annual relapse rate, worse medullary symptoms and disability, increased incidences of brain lesions and LETM, and was frequently associated with thyroid diseases [137]. Interestingly, patients who had medullary involvement more often had headaches, neuropathic pain, and a movement disorder compared with other NMOSD patients without medullary involvement [137].

#### 6.2.2. Oculomotor Abnormalities

A number of oculomotor manifestations, similar to the ones described in MS, have been observed with both AQP4- and MOG-antibody associated NMOSD including (1) walled-eyes bilateral internuclear ophthalmoplegia (WEBINO) associated with a midbrain tegmentum lesion adjacent to the aqueduct on brain MRI [138,139], (2) ocular oscillations, including up-beating, down-beating, central vestibular nystagmus, and opsoclonus myoclonus syndrome, [140], (3) nuclear [141] and bilateral trochlear nerve palsy [142], and (4) central Horner syndrome [143], which has occasionally been described in MS in relation to brainstem lesions [144,145,146]. Thus, with overlay in brainstem symptomatology between MS and NMOSD, MRI of the brain and spinal cord, serology, and most importantly, high index of suspicion are expected to lead to the final diagnosis.

#### 6.2.3. Other Atypical Brainstem Presentations

**Excessive yawning** unrelated to sleep deprivation or fatigue was reported in nine patients with the MOG antibody; five out of nine patients had yawning as a presentation of the illness in association with nausea, vomiting, and hiccups. The duration of this excessive yawning lasted two to 16 weeks. The MRI results were abnormal in all patients with brainstem and hypothalamic lesions [147].

**Encephalopathy**, **albeit not a classical symptomatology of brainstem disease and NMOSD**, has been associated with diencephalic and brainstem involvement and confused with Wernicke’s encephalopathy. The confusion between the two entities extends to the histological level, particularly considering that the hallmarks of Wernicke’s encephalopathy are periventricular involvement of thiamin-metabolism-rich areas with cytotoxic edema of astrocytes and neurons and hemorrhage [148]. While a new onset encephalopathy with focal symptoms and demyelination on CNS imaging is evocative of ADEM or Susac’s syndrome [149,150], encephalopathy presentation in an established case of NMOSD should trigger the search for posterior reversible encephalopathy syndrome, a treatment complication and more recently overlap syndrome or NMOSD-encephalitis complex that will be discuss later in this review. Subsequent relapses can hint at this diagnosis.

## 7. Tumefactive Demyelinating Lesion Pattern Recognition: From Clinically Isolated Syndrome to MS, NMOSD and Others

### 7.1. Multiple Sclerosis-Associated Tumefactive Demyelinating Lesions (MS-TDLs)

TDL, defined as solitary lesions ≥2 cm, might herald symptoms of MS and represent a diagnostic challenge when occurring in an isolated manner. Given et al. reported a pictorial essay that summarized the MRI appearance of TDLs [151]. TDLs tend to be well delineated with minimal mass effects and edema. TDLs typically occur at the supratentorial level, centered in the white matter, with or without extension into the cortical gray matter. Fifty percent of TDLs typically enhance in an incomplete ring pattern, with the open side facing the cortex. Several studies reported a centrally dilated vein and decreased perfusion in comparison to tumors and normal-appearing white matter [152,153]. The presence of centrally dilated veins within TDLs was again confirmed using ultrahigh field 7T MRI of the brain [154,155]. There have been steady attempts to differentiate TDLs from brain neoplasms through locating a novel combination of imaging techniques that allow clinical rather than surgical diagnosis. For example, Mabray et al. demonstrated that TDLs can be diagnosed with a high degree of specificity and differentiated from high-grade gliomas and primary CNS lymphoma on preoperative MRI by using a combination of criteria including incomplete rim enhancement, the presence of multiple lesions, and high minimal apparent diffusion coefficient values on brain MRI [156]. Other authors used conventional and non-conventional imaging techniques to differentiate brain tumors from TDL including ^11^C-methionine positron emission tomography (MET-PET) [157], magnetic resonance spectroscopy, and conventional angiography. In addition to vessel-like structures on TDLs, multiple venous dilatations around TDLs based on angiography can be useful for the diagnosis of large TDLs [158]. Others have attempted to differentiate TDLs from high-grade glioma using cerebral blood volume (CBV) and flow (CBF), calculated from dynamic contrast enhanced perfusion MRI. Perfusion MRI of regional CBV and CBF were reduced among demyelinating patients [159]. An additional challenge of TDL is the possible association of TDL(s) and tumors. This is illustrated by a case of a tumefactive demyelinating MS and an anaplastic oligodendroglioma where the MRI of a patient’s brain fulfilled Barkhof’s criteria, and the CSF study was abnormal with the presence of oligoclonal bands. An ^18^F-FDG-PET scan was performed that demonstrated increased tracer uptake, as expected with a brain tumor and brain biopsy showed an anaplastic oligodendroglioma [160].

Another challenging scenario is the association of primary CNS lymphoma and TDLs both demonstrating the same location predilection and steroid responsiveness. Primary CNS lymphoma manifests as a uniformly contrast-enhancing mass with predilection to periventricular and superficial locations, often contacting ventricular and meningeal surfaces. The lesions are hypo- or isointense on T2-weighted imaging and have prominent perilesional edema. The presence of a mixed iso- and hyperintense lesion on T2, the lack of cortical involvement, and mass effect are in favor of TDL. A computed tomography (CT) scan of the brain demonstrates hypoattenuation in TDL and hyperattenuation in lymphoma, underlining the importance of combining imaging modality with CT and MRI. In both pathologies, magnetic resonance spectroscopy demonstrates increased lipid, choline/creatinine, and myoinositol, and decreased N-acetylaspartate peaks, but elevated glutamate/glutamine peaks favor TDL. Serial MRIs have shown continuous evolution with TDL and stability of the content of the neoplasm [161]. Long-term evolution of an isolated TDL is unknown and limited by the duration of the follow-up. However, like any clinically isolated syndrome, a group will get disseminated in time and space evolving into MS or NMOSD; a second will remain stable for the duration of follow-up, and a third might evolve into a different diagnosis [162]. Lastly, TDLs have been reported [163] with fingolimod use in MS and inadvertently in NMOSD, fingolimod discontinuation [164,165,166] or de-escalation from natalizumab [167,168,169]. These situations might pose a diagnostic challenge in case of lack of familiarity with these scenarios. Albeit an uncommon problem, TDL might represent an investigation challenge prior to, and following the diagnosis of MS [170]. Again, ultrahigh field 7T brain MRI might be promising in tumefactive demyelinating from non-demyelinating lesions [171].

### 7.2. Neuromyelitis Optica Spectrum Disorder-Associated Tumefactive Demyelinating Lesions (NMOSD-TDL) and Hemispheric Presentations

Extensive hemispheric lesions in areas that are not enriched with AQP4 is a pattern described in NMOSD [24,172,173]. A priori, the term tumefactive demyelinating lesion (TDL) evokes the diagnosis of MS. However, a Korean study followed 31 patients with at least one TDL over a mean period of 37.6 months. During this observation period, 11 patients remained idiopathic (six had a single event, and five had recurrent demyelinating disease inconsistent with MS or NMOSD), 11 patients developed AQP4-NMOSD, seven evolved into MS, and two had an alternative diagnosis. The increased conversion of TDL to NMOSD in this cohort could be due to the ethnicity of the studied population, but prior reports on TDL did not systematically test for the AQP4 antibody [162]. A common MRI pattern of TDL-associated NMOSD includes T2-high and T1-iso-to-hypointense lesions, increased diffusivity on apparent diffusion coefficient map, and hypo- or isointensity on diffusion-weighted images or hyperintensity, probably due to T2 shine-through. Contrast-enhancement is typically absent or faint, an indication of the integrity of the BBB [173]. However, in the absence of other clinical, paraclinical, and imaging findings, the presence of TDL with partial ring enhancement could be easily confused with tumefactive MS [174]. Magnetic resonance spectroscopy of six TDLs in three patients with NMOSD showed increased Cho/Cr and decreased N-acetylaspartate peaks/Cr ratios in all of the patients and a lactate peak in two [175]. Posterior reversible encephalopathy syndrome with supratentorial and asymmetric hemispheric presentation has been reported with NMOSD [172,176]. The clinical presentation of TDL-associated NMOSD and posterior reversible encephalopathy syndrome-associated NMOSD is somewhat similar, with a variable degree of encephalopathy and focal symptoms such homonymous hemianopia [172,176].

### 7.3. The relationship of Balo’s Concentric Sclerosis to TDL, MS and NMOSD

**Figure 6:**
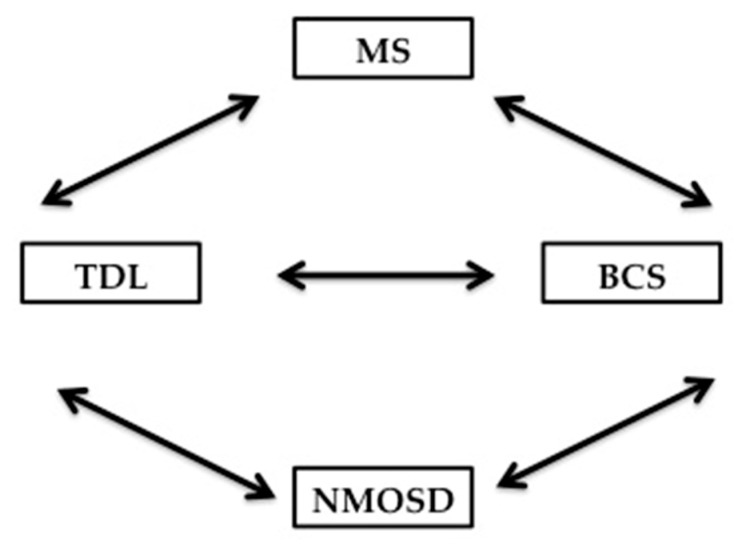
Diagram summarizing the relationship between TDL, BCS, MS and NMOSD; TDL: Tumefactive demyelinating lesion; BCS: Balo’s concentric sclerosis; MS: Multiple sclerosis; NMOSD: Neuromyelitis optica spectrum disorder.

Balo’s concentric sclerosis lesion, which is not the focus of our review, falls under the category of atypical demyelination and is characterized radiologically and pathologically by concentric rings of demyelination and remyelination. Pathologically, the concentric configuration of the lesion is explained by the presence of radially oriented cytokines gradient that provide Balo’s lesion at the edge with some preconditionning to ischemia and less demyelination. This is supported by autopsy studies confirming upregulation of hypoxia-inducible proteins [177]. BCS lesions can be confused with TDL becasuse of their large size, particularly when the layering is not easily discernible, but multiple Balo’s lesions can coalesce to form a TDL radiographically. The evolution of a TDL into a BCS has also been reported in the literature [178]. However, the relationship between BCS, MS and NMOSD has not been clearly defined. Knowing that demyelination is the common denominator between these 4 entities, there are unique characterististics for TDL and BCS that differentiate them from MS and NMOSD [179]. Like TDL, BCS can evolve into MS or NMOSD; conversely, lesions of the Balo’s type can be seen in MS and NMOSD [180,181]. Ultrahigh field 7T MRI of the brain holds promise in potentially differentiating MS from NMOSD, TDL from non-demyelinating ones, and prognosticating which CIS might evolve into MS versus not based on the visualization of the central vein sign [154,171]. The interdependent relationship between BCS, TDL, MS & NMOSD is summarized in Figure 6.

## 8. Clinical Spectrum of MOG-Antibody-Associated-Inflammatory Demyelinating Disorders

### 8.1. Neuromyelitis Optica Spectrum Disorder-Associated Myelin Oligodendrocyte Glycoprotein Antibody (MOG-NMOSD)

Until recently, MOG-associated NMOSD has been occluded by its grouping with the AQP4-antibody seronegative group. Currently, the diagnosis of seronegative NMOSD is made with at least two clinical core criteria meeting certain specific requirements, as recently reported [42]. With overlap in clinical and MRI presentation of AQP4-associated NMOSD and MOG-associated NMOSD, it is necessary and important to determine a pattern to differentiate between the two conditions. Moreover, the condition of MOG-NMOSD is not yet widely recognized due to the absence of commercially available tests. Additionally, as of today, there is no gold standard for the optimal MOG-antibody assay. The largest two series of MOG-NMOSD studies were reported around the same time by the following two groups: one from Germany (*n* = 50 patients), referred to as the “German series”, by the NEuroMyelitis Optica Study group (NEMOS) and one from Spain (*n* = 56 patients) referred to as the “Spanish series” [22,43]. The findings from these two studies are generally representative of what is reported in the literature to date on MOG-NMOSD and are comparatively summarized in Table 3. The two studies share some striking similarities in the mean/median age group, with wide age range, concordant with other studies including Chinese Han patients [83,108]. Females are affected more often, as observed for MS and by other studies [182], but different from the 1:1 or even male gender dominance reported by others [108]. Unlike AQP4-NMOSD, there was no female gender skewing. Almost all of the patients were White in both series. The median duration of follow-up was longer in the German study, but the range was similar in both studies. The number of relapses was greater in the German study. In both series, optic neuritis was the most common initial symptom in 60–64% of the patients in isolation and 70–74% in combination, reflecting the increased expression of MOG in the optic nerve compared to other CNS areas [108,183]. Importantly, the presentation of bilateral simultaneous optic neuritis or optic neuritis with transverse myelitis is common in MOG-NMOSD and greater than that seen for AQP4-NMOSD, which invites a careful search of the diagnostic criteria, as previously reported [108,184]. The German group reported the time duration for the second attack to be five months, but importantly, presentation of the second attack was very similar to the first attack. Indeed, 72% of patients who presented with optic neuritis had recurrent optic neuritis, and 76% who presented with transverse myelitis had recurrent transverse myelitis. A total of 71% and 81% of the patients in both Spanish and German series, respectively, had a relapsing course, which was greater than that reported by other groups who had a shorter duration of follow-up [55,108]. The clinical presentation at last follow-up was not presented similarly in both studies. At last follow-up, the disease was disseminated in 32% of patients (18 of 56) in the Spain series and 44% of patients (22 of 50) in the German series. The difference is likely due to the longer disease duration in the German series (75 months vs. 43 months in the Spanish series). While the absolute number of relapses was higher in the German group, the annual relapse rate was within range (equaling 0.8 for the isolated optic neuritis group and highest at 1.17 for the ON-TM combination group). The final outcome in both series differed, and was worse in the German series. A total of 37% of patients (14 of 38) were functionally blind or had severe visual loss in at least one eye, defined as 20/100 < visual acuity ≤ 20/40. Weakness or ataxia was the etiology of gait impairment in 25% of the patients. In the Spanish series, 19% of those affected by optic neuritis had severe vision loss, defined as visual acuity < 0.1%, and 11% had a moderately severe-to-advanced disability (Expanded Disability Status Scale (EDSS) 4–7). There was one case of death in the German series due to brainstem encephalitis with secondary respiratory failure. Explanatory factors for these discrepancies might include differences in ascertainment of the cases and definition of deficit, and genetic and environmental factors that are known to somewhat modify the risk for autoimmune diseases (such as sun exposure, vitamin D level, diet, etc.). Also likely at play are longer disease duration with a cumulative effect on the CNS from repetitive relapses. Despite the worse outcomes in the German group, comparison of recovery rates from optic neuritis and/or transverse myelitis attacks to previously published rates for AQP4-NMOSD showed an improved recovery rate (more complete and less partial/no recovery) [22], but less than observed with MS [109,185]. Both studies reported serum antibody titers; however, the German group provided more information on the profile of the antibody as a function of the clinical presentation, relapsing or remitting status, and in response to treatment. For example, in the German study, antibody titers were variable from 1/160 to 1/20,480, with the highest titers found for the combination of optic neuritis-transverse myelitis and the transverse myelitis groups at last follow-up. This trend was also seen in the Spain series. Serum antibodies sampled within few days to three months of disease onset were positive in all patients and persistent in the serum up to 10 years for available samples of this duration. The greatest antibody titers were observed within 14 days of the attack onset and occasionally during disease remission. During disease exacerbation, the titers were variable intra- and inter-individually. Serum antibody titers equally decreased during disease remission, following relapse treatment with corticosteroids and plasma exchange, and maintenance treatment with immunosuppressants, a decrease reflective of the natural remission rather than treatment [51]. Decreased MOG antibody titers were observed during rituximab therapy, and MOG antibody titers increased with B-cells recurrence. MOG antibodies in the CSF were present in 12 of 15 patients tested during disease exacerbation and decreased during remission [51]. Greatest CSF MOG antibody titers were observed for transverse myelitis. To determine antibody class and sub-class, the serum and CSF of eight seropositive patients during exacerbation and six in remission were analyzed. All of the patients had positive MOG-IgG1 in the serum and in the CSF [51]. Two out of the 20 patients tested from the MOG antibody group were positive for IgM in the serum only; no CSF or serum tested positive for MOG-IgA [22]. Overall, the CSF profile of MOG-NMOSD mirrors the profile of AQP4-NMOSD. Like AQP4-NMOSD, neutrophils and granulocytes were present in the CSF in about two-thirds of the cases. Consequently, the absence of oligoclonal bands and/or the presence of granulocytes and neutrophils in the CSF should challenge the diagnosis of MS. MRI of the brain, brainstem, spinal cord, and orbits in MOG-NMOSD are reminiscent of that seen in AQP4-NMOSD, except for the differences noted in Table 3. For the brain MRI, supratentorial abnormalities were seen at onset in 35.4% of patients, and infratentorial was seen in 14.6% of the patients [51]. Supratentorial lesions were periventricular, involving the corpus callosum in a confluent manner in the frontal, parietal, temporoparietal, and occipital deep white matter. Some lesions were subcortical or juxtacortical, including the insular cortex. Leptomeningeal enhancement and basal ganglia involvement were observed in 1 in 50 patients each. Infratentorial lesions at onset involved the cerebral peduncles, pons, medulla (particularly the area postrema), the periaqueductal gray matter, and the cerebellar hemisphere and peduncles. Corpus callosum lesions were longitudinally extensive as seen with patients with AQP4 antibodies [23]. Most importantly, 50% of the patients fulfilled the McDonald 2010 criteria by MRI, and 62.3% fulfilled the 2006 NMO criteria. Barkhof’s criteria for DIS were fulfilled in 15% of the cases that were positive MOG antibody and had a history of transverse myelitis and/or optic neuritis, and in 26.9% of the cases that were positive for the MOG antibody and had a history of brain lesions [51]. Visual evoked potentials demonstrated subclinical optic nerve damage, and asymptomatic lesions were present in the brain, cerebellum, or spinal cord, which is a pattern observed in MS but not in AQP4-NMOSD. Importantly, the presentation of bilateral simultaneous optic neuritis or optic neuritis with transverse myelitis is common and invites a careful search for the correct diagnosis, as reported by others [108,184]. Typically, optic neuritis patients had improved recovery compared to transverse myelitis patients. Deterioration was observed after corticosteroid withdrawal, as described by others [61,186]. Treatment with plasma exchange and immunoadsorption, with or without steroids, has been associated with complete recovery in about one-third of the cases, and partial recovery has been observed in the majority of the cases. There were 14 untreated attacks, two without recovery, with one being fatal, three with partial recovery, and nine with complete, or almost complete, recovery [22].

### 8.2. Pediatric Acute Disseminated Encephalomyelitis (ADEM)

Few studies demonstrated an increase in serum anti-MOG antibodies in patients with MS compared to controls. The prevalence and titers of MOG antibodies in the studies were low and had no predictive or prognostic significance in MS [187]. Subsequently, anti-MOG antibodies were identified in pediatric demyelinating diseases, including ADEM and clinically isolated syndromes. The presence of MOG antibodies correlated with a younger age of onset and the initial clinical presentation of ADEM. Additionally, increased titers correlated with an initial presentation of ADEM rather than clinically isolated syndrome. Most children with MOG antibody-associated ADEM (i.e., MOG-ADEM) had a rapid decline in MOG antibody titers with recovery and persistence with incomplete recovery. Based on the MRI of the brain and spinal cord, these children had more fluffy lesions and LETM with complete resolution and improved outcome [49,50].

An interesting syndrome associated with MOG-ADEM in children is the ADEM- optic neuritis (ADEM-ON or ADEMON) or multiphasic disseminated encephalomyelitis (MDEM-ON) complex. Table 4 summarizes the 2 cases-series reported in the literature. The natural history and prognosis for ADEM/MDEM-ON remains unknown considering that the median follow-up years was 4–6 years in the 2-case series [188]. Another case of ADEM followed by optic neuritis 71 days later and positive MOG antibody in the serum was reported in a five-year-old Japanese girl [189]. Adding to the list of cases of MDEM-ON is the case of a 4.5-year-old Malyasian girl with MDEM followed by optic neuritis and positive MOG antibodies with relapses triggered by viral infections and a gluten rich diet [190].

Extending the spectrum of MOG-antibody inflammatory demyelinating diseases, Baumann et al. reported a case series involving eight children, six White and two Asian, with MDEM and who were positive for the MOG antibody. These children were followed for four years. The mean number of relapses was three, (ranging from 2 to 4), and the mean inter-attack interval was four months (ranging from one to 48 months). All children had at least two attacks separated by at least three months. Initial multifocal deficit with and without encephalopathy were followed by optic neuritis in some children or MDEM-ON. MOG antibodies were present in all children, but none had theAQP4 antibody. Initial and repeat CSF studies were similar to ADEM-ON and NMOSD. MRI of the brain was characterized as hazy and with TDLs that improved over time, along with cortical gray matter involvement in seven patients, an area rarely involved in AQP4-NMOSD [23]. Asymptomatic white matter lesions were seen in one child. Two LETM and two SSTM were present in four children [191].

### 8.3. Overlap Syndrome or Complex Neuromyelitis Optica Spectrum Disorder Encephalitis

**Figure 7:**
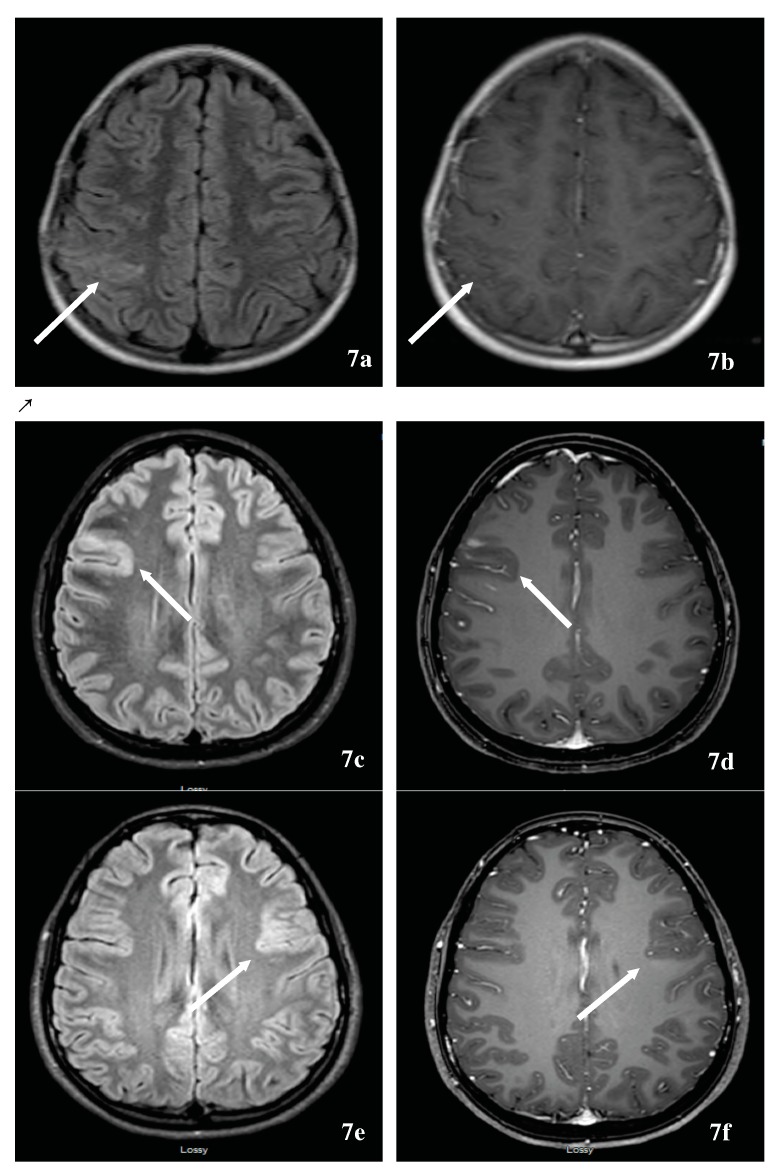
This is the case of a 21-year-old woman who presented with a history of bilateral optic neuritis at the age of 10. She subsequently had recurrent encephalitis with cortical swelling and seizures at the age of 13, 17 and 19. Axial FLAIR shows a right parietal cortical T2 hyperintensity with swelling (**7a**) associated with minimal T1 hypointensity without contrast enhancement (**7b**). Recurrent encephalitis was associated with a new right frontal cortical T2 hyperintensity with swelling (**7c**) associated with minimal T1 hypointensity with contrast enhancement. (**7d**). A third episode demonstrated a new left frontal cortical T2 hyperintensity with swelling (**7e**) associated with minimal T1 hypointensity with contrast enhancement (**7f**). Serum MOG-antibody was positive.

In this review, Table 5 summarizes all of the cases of NMOSD associated with encephalitis (*n* = 46) reported in the literature from 2010 onward [192,193,194,195,196,197,198,199,200]. While the authors of these case reports or series aimed to report clinical cases with “overlap syndrome”, the series by Hacohen et al. was a systemic evaluation of CNS autoantibodies in pediatric demyelination syndrome including ADEM, optic neuritis, LETM, and NMO [194]. The age range for the entire group as shown in Table 5 is broad, from 16-months- to 65-years-old. The clinical presentation was of encephalitis, abnormal movement disorders, unilateral or bilateral optic neuritis, transverse myelitis, seizures, or a combination of the above symptoms. Abnormalities on imaging were variable from normal MRI brain to multifocal white matter lesions and LETM, multiple cranial nerve enhancement, and gray matter involvement (e.g., cortical and basal ganglia). Some of these cases were associated with positive serum AQP4-IgG, others were associated with the MOG antibody, and in the majority of the cases, whenever done, CSF NMDA-R antibody was positive. Serum NMDA titers were not available for the majority of cases, except in one study. Glycine receptor and voltage-gated potassium channel antibodies were positive in one and three cases, respectively [194]. In some case, antibodies for both NMDA-R and MOG were exclusively present in the CSF. CT scan of the chest, abdomen and pelvis were performed in a number of cases and were negative, except in one case of ovarian teratoma [199]. Pleocytosis in the CSF was frequently present with protein elevation. Oligoclonal bands were present in 13 cases, absent in 18 cases, and not available for the rest. Full recovery was reported in 10 cases, incomplete recovery in 21 cases, unchanged in two patients, death in one case due to rapidly evolving pneumocystis pneumonia, and data was not available for 12 cases. Acute management included multimodality treatment with corticosteroids, plasma exchange, and intravenous immunoglobulin in different combinations. Immunosuppressants, such as rituximab [197,198,200], mycophenolate mofetil [197,198], and azathioprine [199], were used mostly on acute basis, but for an unknown period of time. We recently had a referral for diagnosis and treatment of a 21-year-old woman with history of bilateral LEON associated with perineural sheath thickening and optic nerve twisting on orbital MRI at the age of 9 (case not published). Throughout the years, she had three more episodes of cerebral encephalitis (see Figure 7) associated with cortical swelling on brain MRI and seizures at the age of 13, 17, and 19. A MOG-antibody associated disease was suspected. Extensive serum and CSF autoantibodies testing was positive for serum NMDA-R, thyroid peroxidase, voltage gated potassium channel autoantibodies at low titers and for MOG-antibody.

Upon literature review, we found that she had an overlap syndrome reported as benign, unilateral, cerebral cortical encephalitis with epilepsy associated with the MOG-antibody, recently reported in four men, aged from 23 to 39 years. All of these men had seizures, and three had associated encephalopathy and/or psychosis. Unilateral optic neuritis (two cases) and seizures (one case) were observed at seven months and 35 months prior to encephalitis. Encephalitis, seizure, and optic neuritis were coincident in one case [192]. Our case was different, as following an initial episode of bilateral LEON, she had three more episodes of encephalitis and seizures associated with waxing and waning of cortical T2 hyperintensity over the course of 10 years. She was very recently initiated on Rituximab, and remains on lamotrigine for seizure management, although she has not had any seizures in isolation of encephalitis. In summary, the number of overlap syndrome cases might be underestimated. With the lack of familiarity with this novel entity, it is perceivable that cases of NMOSD-encephalitis complex are misdiagnosed as ADEM, paraneoplastic syndromes or toxic metabolic encephalopathy, to cite a few.

### 8.4. The Myelin Oligodendrocyte Glycoprotein (MOG) Antibody and Its Association to Other Autoantibodies

The association of the MOG antibody in inflammatory demyelinating diseases to other autoantibodies (e.g., thyroid, celiac, antinuclear antibodies, Sjogren’s, glomerular basement membrane) has been reported in several papers [22,43,53,55], but this association is less commonly found in comparison to AQP4-NMOSD. Double seropositivity of AQP4 and MOG antibodies in NMOSD and its limited forms was found by ELISA techniques [201], by cell-based assays [62,98,202], and in a single patient with gastric cancer and NMO [53]. Known to be rare, these cases present clinically with severe deficits because of recurrent optic neuritis or bilateral optic neuritis and simultaneous transverse myelitis. The frequency of AQP4, glycine receptor alpha 1 subunit, and MOG antibodies was determined in a cohort of patients with isolated optic neuritis; the combination was found in 45% (23 of 51) of the cases of the cases and was associated with unilateral or bilateral, severe or recurrent optic neuritis [203].

## 9. Other Autoantibodies, Diseases, and Biomarkers Associated with Neuromyelitis Optica Spectrum Disorder

### 9.1. Aquaporin 1-Antibody Associated with NMOSD (-NMOSD)

As previously noted, there are at least 12 related AQPs expressed in mammalian tissues, and a greater number of homologues are expressed in plants and lower organisms. Both aquaporin 1 (AQP1) and APQ4 are expressed in astrocytes, reflecting some redundancy that might functionally important [33]. The differential expression of AQP1 and AQP4 in different parts of the astrocytes has been demonstrated in the gray and white matter [204]. In an attempt to determine if AQP4-seronegative NMOSD had other associated antibodies, a group of researchers from Greece analyzed the sera of patients with inflammatory demyelinating diseases, other neuroimmune diseases and healthy control for the presence of AQP1 antibodies. A total of 348 sera referred for AQP4-IgG testing were analyzed. Antibodies to AQP1 and AQP4 were analyzed using radioimmunoprecipitation assay (RIPA) and two-steps RIPA, a more sensitive technique. Aquaporin 1 antibodies could not be detected by cell-based assays. A total of 42 out of 348 sera (12%) tested positive for the AQP4 antibody using RIPA, and 44 out of 306 remaining sera (14%) tested positive for the AQP1 antibody. A total of 14 out of 42 sera (33%) were double positive for the AQP4 and AQP1 antibodies, meaning there were a total of 58 out of 306 patients (19%) who had the AQP1 antibody. None of the MS, neuroimmune diseases or healthy control samples had the AQP1 antibody. The female-to-male ratio was 2:1 for the AQP1 antibody and 10:1 for the AQP4 antibody. Although both AQPs share amino acid sequence identity (51%), antibodies in the double-positive sera did not cross-react with the other antigen. Most AQP1 antibodies were more often bound to the extracellular portion of the AQP1 loop A compared to loop C. Loop A is the more antigenic portion of AQP, has reduced identity, and shows homology to the corresponding AQP4 sequence. Most APQ1 antibodies were IgG1. The double-positive sera, however, had high antibody titers, indicating an interdependent immune system triggering mechanism. Epitope spreading across the AQPs in the double-positive group is also a possibility. The clinical and imaging data of a cohort of 22 patients, 17 with NMOSD and five with MS were reviewed. A total of 16 out of these 22 patients with imaging data had LETM, one had transverse myelitis only, and two had MS with a dominant spinal load. Five out 16 patients had LETM and optic neuritis. Three out of the 22 patients had classic MS and were positive for the AQP1 antibody. However, the antibodies in the MS sera were different and could not bind the extracellular portion or the whole AQP1 antigen. Interestingly, these three patients had neoplasms, thus raising the possibility that the antibodies were triggered by some neoplastic antigens. The authors concluded that the role of AQP1 in NMOSD remains unknown [205]. Findings in this study were not replicated by two more studies [206,207]. AQP1 antibody was tested in a group of 249 patients with different types of inflammatory demyelinating diseases, using CBA with Triton X-100. There were 98 AQP4-antibody-positive and 151 antibody-negative serum. A total of 73 out of 98 serums (74.5%) were positive for AQP1, meaning 73 were double positive. A total of 49 out of 151 AQP4-antibody-negative cases turned to be AQP1-antibody-positive cases, and these had relapsing optic neuritis, LETM, MS, or NMO. The authors concluded that there was some limited value in using the AQP1 antibody when the AQP4 antibody was negative. Interestingly, and in keeping with the prior group’s findings [205], CBA without the use of Triton X-100 had a low efficiency for detecting the AQP1 antibody. However, adding Triton X-100 resulted in a dramatic increase in AQP1 antibody detection/determination. Triton-100 is a detergent commonly used experimentally to permeabilize the membrane of living cells and increase antigen retrieval [207]. A third group, Sanchez Gomar et al., attempted to detect AQP1 antibodies using CBA and ELISA. The AQP1 antibody was undetectable using CBA and detected with low titers by ELISA in few samples. The conclusion was that the study did not allow a sustained detection of anti-AQP1 in serum of NMOSD patients analyzed by CBA or ELISA [206]. This was further confirmed by the absence of AQP1-antibody using a live CBA in a cohort of patients with NMOSD, MS and controls [208]. Thus, AQP1 antibody does not convincingly appear to be a biomarker for NMOSD. Nevertheless, the quest for more autoantibodies to diagnose or prognosticate inflammatory demyelinating diseases will continue. The story of AQP1 antibody is not unique and the inward rectifying potassium channel 4.1 (KIR 4.1) is another antibody where different groups across the world had conflicting results [209,210,211,212,213,214]. KIR 4.1 is a membrane protein expressed by oligodendrocytes, a subset of astrocytes and various tissues such as kidney. An editorial by Hemmer provides a comprehensive analysis of the problem of isolating autoantibodies in human diseases. Detection of autoantibodies by protein based assays remains the winner but even then, posttranslational modification of the protein needs to be monitored during the assay [215].

### 9.2. Neuromyelitis Optica Spectrum Disorder as a Paraneoplastic Syndrome

Incidental malignancies were found in 31 patients among 180,000 patients evaluated for paraneoplastic antibodies and included breast, lung, thymic, uterine cervix, B-cell lymphoma, monoclonal gammopathy, thyroid Hurthle cell, carcinoid and pituitary somatotropinoma. These malignancies preceded or ensued NMOSD [216]. Treatment-resistant AQP4-LETM significantly improved, and autoantibody response reverted following breast cancer treatment. Screening for malignancy was thus suggested in treatment-resistant demyelinating disorders [217]. A case of invasive, poorly differentiated breast ductal carcinoma was reported in a 29-year-old woman who presented with brainstem symptomatology. Because of a prior history of cardiac surgery, brain MRI was not possible, but a PET scan demonstrated increased tracer uptake in the brainstem, medulla, pons, dorsal midbrain, and left medial temporal lobe, which was consistent with brainstem and limbic encephalitis. Serum and CSF were positive for the AQP4 antibody. The patient had a modified radical mastectomy followed by plasma exchange (PLEX) and rituximab with significant recovery at three months [218]. Paraneoplastic NMOSD has been also reported in association to a metastatic carcinoid tumor to the liver. While the tumor antedated the neurological manifestation by six years, hepatic metastases were coincident. Interestingly, AQP4 cells were present interspersed between neuroendocrine tumor cells on pathological examination of the metastases [219]. Similar findings of thyroid cancer expressing AQP4 were reported in a patient with thyroid cancer, predating the onset of NMOSD [220]. Negative AQP4-antibody NMO-like disorders have been associated with collapsing response-mediated protein 5 (CRMP5) and different malignancies such as small cell lung, prostate, thyroid papillary, and renal cancer, and thymoma [221] as well as anti-amphiphysin antibody and invasive poorly differentiated breast adenocarcinoma [222]. The association of the MOG antibody inflammatory demyelinating diseases to tumors was reported in the MOG-NMOSD German series; there was one case of mature cystic teratoma and a ganglioneuroma in one patient. Otherwise, there were no other cases of malignancy [22].

### 9.3. Other Biomarkers Associated with Neuromyelitis Optica Spectrum Disorder

As diagnostic biomarkers, AQP4 and MOG antibodies allow for improved tailoring of maintenance therapy. In a sense, they are also prognostic biomarkers as MOG-inflammatory demyelinating disease appears to be less aggressive than AQP4-antibody-associated diseases. The following section addresses several available biomarkers relevant to the diagnosis and prognosis of MOG-inflammatory demyelinating diseases and AQP4-antibody-associated diseases and allow differentiation of the two conditions. This is by no means an exhaustive list, and the reader is directed to the following update on NMO biomarkers [223].

#### 9.3.1. Cerebrospinal Fluid Myelin Basic Protein (CSF MBP)

Ikeda et al. [224] analyzed MBP and GFAP levels in patients with MOG-NMOSD patients with the MOG antibody [224]. During an acute attack, CSF MBP was increased by 10-fold, but CSF and GFAP was undetectable. Similar work was reproduced in a larger multicenter international collaborative study where MOG and AQP4 antibodies and markers of damage myelin (i.e., MBP) and astrocytes (i.e., GFAP) were evaluated in the CSF of patients with NMOSD and MS. A third of the NMOSD patient sera were positive for the MOG antibody and two-third were positive for the AQP4 antibody. Three-fourths of the patients with NMOSD had either MOG- or APQ4-antibody in the CSF; again, one-third of the patients were positive for the MOG antibody, and two-thirds were positive for the AQP4 antibody. CSF GFAP was elevated in the AQP4-antibody-positive patients but not in the MOG-antibody-positive and MS cases. Elevated CSF MBP was observed in both MOG- and AQP4-positive antibodies, and both groups exhibited higher MBP levels compared with the MS group. Myelin damage in AQP4-antibody-positive patients with NMOSD is recognized to be secondary to antibody-dependent and complement-dependent astrocytic injury. The direct binding of MOG antibodies to MOG causes myelin damage and/or oligodendrocyte dysfunction. Pro-inflammatory cytokines released during attacks may induce the recruitment of immune cells (MOG-specific reactive T-cells and B-cells, macrophages, etc.) and the release or synthesis of other cofactors promoting demyelination [225].

#### 9.3.2. Cerebrospinal Fluid (CSF) Glial Fibrillary Acid Protein (GFAP) and S100

Glial fibrillary acid protein (GFAP) is a monomeric intermediate filament protein in the astrocytes. A marked increase in GFAP in the CSF of AQP4-antibody-positive NMO is supported by the work of Misu et al. [226]. CSF-GFAP values in the AQP4-antibody-positive group were increased by 10,000-fold compared with the MS and ADEM groups and by 20-fold in the spinal cord infarct group. Treatment of three NMO patients with corticosteroid remarkably decreased CSF-GFAP close to normal levels. The significant increase of CSF-GFAP is supported by pathological studies showing a loss of GFAP and AQP4 reactivity in the acute perivascular lesions [31,227]. In MS, mild CSF-GFAP elevation likely results from reactive astrogliosis in chronic lesions, whereby GFAP is released into the CSF. In NMO, the CSF-GFAP levels strongly correlated with EDSS and spinal lesion length (*r* > 0.9 for both correlations). The CSF-GFAP levels were reportedly high in other destructive pathologies such as stroke and herpes encephalitis, but the levels were still 100-fold less than NMO. The CSF levels of S100-B, expressed by astrocytes ensheathing blood vessels and by glia (microglia and oligodendrocyte), were also analyzed. During acute NMO exacerbations, CSF-S100B levels were 100-fold greater than MS, ADEM and spinal cord infarction. Like CSF-GFAP, the values decreased after corticosteroid therapy, and the levels strongly correlated with the length of spinal cord lesions (*r* > 0.9). Thus, measuring astrocytic markers, especially CSF-GFAP, would be useful for assessing astrocytopathy and clinical severity of NMO, as well as to discriminate between AQP4-antibody-positve NMOSD and MS [226,228].

#### 9.3.3. Interleukin 6 (IL-6) in Neuromyelitis Optica Spectrum Disorder (NMOSD)

Between all the cytokines and chemokines described with NMOSD, interleukin 6 (IL-6) appears to be the most relevant clinically, as it has shown the strongest correlation with clinical variables, including CSF GFAP, cell counts, and AQP4 antibody titers [229,230] and EDSS [230]. IL-6 has immunologic and non-immunologic roles in the CNS [231]. Parallel to its increase in the CSF, increased serum IL-6 was observed in patients with NMO [229] Further, IL-6 induces the differentiation and maturation of B-lymphocytes into plasmablasts further increasing the production of AQP4 antibodies, and the differentiation of naïve T-cells into TH17 cells. Likewise, TH17 cells produce IL-17 that further increases the production of IL-6. During initial [229] and recurrent NMOSD exacerbations, CSF-IL6 levels increase to the same extent, independent of lesion location. It is not specifically known if IL-6 is released by astrocytes or responsible for their injury, but an autocrine function of IL-6 has been reported [232]. The increase in serum and CSF-IL-6 has been therapeutically exploited, allowing for treatment of NMOSD with tocilizumab, a humanized monoclonal antibody inhibitor of IL-6 that is described below [229,233,234].

## 10. Tips on Management of Inflammatory Demyelinating Diseases of the Central Nervous System (CNS), with a Focus on Neuromyelitis Optica Spectrum Disorder (NMOSD)

### 10.1. Management of Acute Demyelinating Relapses Of The Central Nervous System

Acute management of demyelinating events is similar across the entire demyelination spectrum and consists of high doses of intravenous methylprednisolone (IVMP) and plasma exchange (PLEX) for severe demyelinating attacks of the CNS [235]. High-dose IVMP did not change the six-month visual acuity in the treated vs. untreated groups during an optic neuritis treatment trial [72]. However, corticosteroids are the cornerstone of CRION management, starting with high-dose pulse methylprednisolone followed by a prolonged oral prednisone taper [59]. Timely institution of corticosteroids leads to prompt recovery from pain, but rapid weaning can lead to irreversible damage. This deleterious response to steroid withdrawal should be an admonition to the diagnosis of CRION [59]. Likewise, exquisite steroid sensitivity is observed with granulomatous optic neuropathy and sarcoidosis and both conditions should always be ruled out [57]. Furthermore, corticosteroids are crucial in the management of NMOSD-ON, specifically MOG-ON. Ultrahigh doses of IVMP (2–5 g) have been used in severe cases [236]. Furthermore, while oral prednisone taper following IVMP failed to improve disability from relapses in MS [237], a prolonged prednisone taper is recommended with MOG-ON and CRION considering that a rapid withdrawal might lead to abrupt visual loss [22]. This prolonged oral taper is an intermediate therapy providing segue into chronic management. Additionally, the benefits of PLEX in treating severe demyelinating events have been reported [235]. PLEX is a standard of care for steroid-refractory demyelinating relapses per the European guidelines [238] and level B recommendation by the American Academy of Neurology [239]. Due to loss of proteins during plasma exchange, replacement with albumin or other plasma proteins is required. Immunoadsorption (IA) bypasses this problem and allows for a more selective adsorption of antibodies using a selective adsorbent to the antibody. Immunoadsorption using tryptophan or sepharose-conjugated sheep antibodies to human Ig is not infrequently used in Europe for management of steroid refractory NMOSD relapses [240]. The German Neuromyelitis Optica Study Group (NEMOS) recently conducted a retrospective analysis of 871 attacks and 1153 treatment courses in NMO/NMOSD [236]. While steroids were used with the first round of relapse management in 83.6% cases, PLEX was used in 100% of cases at the fifth round. The frequency of attacks treated with a second, third, fourth and fifth treatment modality was 28.2%, 7.1%, 1.4%, and 0.5%, respectively. Reportedly, the percentage of complete and partial recovery increased and the no recovery decreased with successive treatment courses [236]. Other treatment modalities included 54 identified combination therapies, the most common being IVMP followed by PLEX. Predictors of complete recovery included younger age of onset, PLEX/IA as a first treatment course and complete recovery from a prior attack. On the other hand, transverse myelitis attack was inversely associated with complete recovery. Characteristics such as gender, AQP4 antibody status, time from disease onset to attack, and time since previous attack were not predictors of complete recovery. Importantly, time from attack onset to therapy showed a trend that appeared significant. The NEMOS group retrospectively reviewed different therapies used in the management of MOG-NMOSD. High-dose corticosteroids were partially effective, resulting in ultrahigh doses of IVMP use. Oral prednisone taper and additional PLEX were recommended. Variable response to PLEX was observed, perhaps due to timing of the procedure, MOG antibody titers, number of sessions, intensity of the relapse, the extension and site of the demyelinating event, and optic neuritis vs. transverse myelitis vs. other locations. The exchange sessions varied from three to 11, with relapses observed when the number of PLEX sessions was less than seven. The authors recommended using PLEX as a substitute for ultrahigh-dose IV method prednisolone because of the risk of cerebral venous thrombosis that was reported in one case [22].

### 10.2. Maintenance Therapy of Neuromyelitis Optica Spectrum Disorder (NMOSD)

A detailed overview of maintenance therapy in NMOSD and MS is beyond the scope of this review (Table 6). Today, both AQP4- and NMOSD are similarly treated. Regarding maintenance therapies for MOG-NMOSD, azathioprine was ineffective if used without steroids the first six months of treatment. Methotrexate caused a reduced relapse rate in some but not all patients. Attacks were observed during rituximab initiation, likely due to B-cell activating factor (BAFF) increase [241,242]. It is in the present author’s clinical practice to use 1 g of IVMP a week following the first dose of Rituximab (1 g) as worsening inflammation has been observed first hand clinically and by imaging (personal observation). A rapid relapse rate with B-cell repopulation was noticed underlining the importance of monitoring CD19 cells. Like NMOSD-AQP4, interferon beta caused an increase relapsed rate and glatiramer acetate was not effective in relapse prevention [22]. Fingolimod exacerbated optic neuritis symptoms in a case of MOG-ON within three weeks of medication initiation [53]. With the absence of disease definition and serological markers for NMOSD in the past and the persistence of clinical manifestations overlap to date between MS and NMOSD, empirical clinical experience, resulted inadvertently, in morbidity and mortality using MS disease modifying therapies such as interferon beta, glatiramer acetate, natalizumab and very lately alemtuzumab and dimethylfumarate to treat NMOSD [243,244,245,246,247,248,249]. Promising therapies, however, are emerging. Tocilizumab, an IL6- receptor antagonist, was shown to reduce relapse rate in 8 patients with NMOSD by about 90%. A pragmatic use of the infusion at the dose of 8 mg/kg administered every 4 weeks might improve its performance [250]. A humanized IL-6R neutralizing monoclonal antibody, SA237, designed by applying recycling antibody technology to tocilizumab, is currently being tested in 2 clinical trials [251,252]. Recycling allows SA237 to bind to IL-6 receptor multiple times and slows medication clearance from plasma [253].

## 11. Discussion and Conclusions

A number of practical learning points emerge in this review, which is geared toward the pattern recognition of optic neuritis, transverse myelitis, brainstem/cerebellar and hemispheric TDL-associated MS, AQP4-antibody and MOG-antibody NMOSD, overlap syndrome, and some yet-to-be-defined/classified demyelinating disease all unspecifically labeled under *MS syndrome*. In the case of demyelinating syndrome occurring past the age of 50 and as a broad suggestion, one should suspect AQP4-NMOSD and MOG-NMOSD, although both diseases, particularly MOG-NMOSD, are seen in the younger population. Whenever a man presents with optic neuritis or other demyelinating symptom, the most likely root disease is MS or MOG-NMOSD, particularly considering the overrepresentation of women in relapsing AQP4-NMOSD with a women-to-men ratio of 9:1 [111,254]. Whenever optic neuritis presents in an individual younger than 20-year-old, it is recommended that the MOG antibody and AQP4 antibody levels be checked [74]. The classical teaching has been that corticosteroids speed the recovery of optic neuritis but do not necessarily alter the visual acuity outcome at six months. However, severe visual deficit has been associated with AQP4-ON, MOG-ON, and CRION, thus calling for prompt treatment with high-dose steroids in any new patients with optic neuritis particularly when immediate imaging of the brain is not available. With the broadening spectrum of inflammatory demyelinating optic neuritis, MRI of the orbit is crucial; the positive findings have diagnostic and prognostic implications, and can help tailor treatment. Because the long-term management of these conditions differs significantly, testing for both antibodies in a patient with his or her first optic neuritis relapse and normal MRI of the brain is recommended. The spectrum of MOG-inflammatory demyelinating diseases underlines that female gender is not always overrepresented in autoimmune disorders, and the MOG antibody autoimmune phenotype appears in a considerable proportion of male patients compared to other demyelinating diseases. Although the McDonald criteria should be reserved for patients suspected to have MS and are supposed to be of prognostication value, their use to diagnosing MS is common particularly when the clinical presentation of demyelinating disease is in question. By applying these criteria in clinical practice to cases of NMOSD, there appears to be a significant phenotypic overlap with the 2006 NMO, 2015 NMOSD, and the 2010 McDonald diagnostic criteria. Whether AQP4-inflammatory demyelinating diseases and MOG-inflammatory demyelinating diseases should be broadly categorized under the umbrella of NMOSD remains a subject of debate [3,255]. Clinically, the two conditions share striking similarities with the presence of longitudinally extensive optic neuritis and transverse myelitis, the presence of neutrophils and eosinophils in the CSF, and the absence of oligoclonal bands, despite a different underlying pathophysiology, the first being an astrocytopathy and the second a demyelinating disease. A peculiar pattern for MOG-NMOSD emerges, including clinically, the striking presentation of bilateral simultaneous/sequential optic neuritis with transverse myelitis; a dominant optic neuritis phenotype, with a relapse rate higher than MS or NMOSD, nevertheless an intermediate prognosis between MS-ON and AQP4-ON; a wide age distribution with the pediatric and geriatric population on both ends of the spectrum; a versatile clinical phenotypes that has optic neuritis as a component but does not fulfill MS or NMOSD diagnostic criteria such ADEM-ON [188], MDEM-ON [190,191], benign unilateral cortical encephalitis with epilepsy [192]. Pattern recognition extends to imaging with the presence of cortical lesions on MRI; a LEON with anterior involvement, perineural sheath swelling and optic nerve twisting. The course is less aggressive than AQP4-NMOSD due to the difference in the substrate of attack by the antibody. In MOG-optic neuritis, the antibody attacks the MOG antigen, leading to demyelination. In AQP4-optic neuritis, the antibody is pathogenic against the astrocyte, leading to direct neuronal and oligodendroglia damage. The similarity in clinical phenotype, however, is intriguing, and raises the possibility of a downstream common pathway for damage. Another intriguing finding is systemic autoimmunity, which is less often associated with MOG-NMOSD compared to AQP4-NMOSD. Overlap syndromes or NMOSD-encephalitis complex associated with NMOSD and neuronal antibodies (NMDA-R, VGKC, glycine receptor alpha 1 subunit antibodies) have been reported with both conditions but seem to be more common with NMOSD-MOG. These findings parallel the auto-antigens present in AQP4- and MOG-NMOSD, the first being present in the central and peripheral nervous system and other organs, the latter being restricted to the nervous system. Whether the location of the antigen has a bearing on the presence or absence of systemic autoimmunity remains to be determined. Whether the proper classification of NMOSD should be based on clinical or biological phenotype with the identification of new target autoantigens remains unanswered. However, the nosology of NMOSD might need to be revised. With more antibodies being unraveled, the seropositive/seronegative terminology should be abandoned or modified, as it will become a source of confusion once the MOG antibody testing becomes widely available. We propose using AQP4-NMOSD and MOG-NMOSD for true seropositive NMOSD and undefined NMOSD when an antibody is unknown/not present. This terminology will allow the incorporation of future antibodies in the classification of NMOSD. Lastly, based on a recent article showing the MOG antibody against native MOG in patients with MS [256], a question remains unanswered: whether or not MOG-NMOSD is the nebulous borderland between MS and NMOSD.

## Figures and Tables

**Table 1:** Comparative chart of optic nerve (ON) MRI in inflammatory demyelinating diseases (IDDs) of the CNS. AQP4-NMOSD: Aquaporin 4-antibody Associated neuromyelitis optica spectrum disorder; LEON: longitudinally extensive optic neuritis; MOG-ON: myelin oligodendrocyte glycoprotein antibody associated optic neuritis.

MRI	MS	AQP4-NMOSD	MOG-NMOSD
**Optic nerve (Range)** [74,76,77]	Unilateral Short segment 13 mm (8–36 mm) Median # segments: 1 (1–4 mm)	Bilateral LEON, median length 26 mm (range 14–46 mm) Three segments (1–8) Posterior predominance with chiasma and optic tract involvement Milder swelling than MOG-ON Rare twisting	Bilateral LEON Anterior predominance Perineural sheath swelling Tilting & twisting of the optic nerve

**Table 2:** Comparative chart of optic nerve (ON) and spinal cord imaging in inflammatory demyelinating diseases (IDDs) of the CNS. LEON: longitudinally extensive optic neuritis; LETM: longitudinally extensive transverse myelitis; MOG-ON: myelin oligodendrocyte glycoprotein antibody associated optic neuritis; SSTM: short segment transverse myelitis; #: number.

MRI	MS	AQP4-NMOSD	MOG-NMOSD
**Spinal Cord (Range)** [113,115,117]	SSTM LETM less frequent Dorsolateral lesions (SSTM & LETM)	LETM SSTM less frequent Central cord lesion Complete resolution of lesion Bright spotty lesions Linear lesions	LETM SSTM less frequent Central cord lesion Complete resolution of lesion Bright spotty lesions? Linear lesions

**Table 3:** Comparative chart of the two largest reported series on MOG-NMOSD. * Functional blindness is defined as a person who has to use many alternative techniques to perform tasks that are ordinarily performed with sight that his/her pattern of daily living is substantially altered. Such alternative techniques might include reading a newspaper by listening to it over the telephone or using Braille to read a book. ** EDSS score includes visual functional score system (VFSS); *** BMRC: British Medical Research Council score; a score ≤2, refers to severe paresis; ADEM: acute disseminated encephalomyelitis; CBA: cell-based assay; FU: follow-up; IVMP: intravenous methylprednisolone; LETM: longitudinally extensive transverse myelitis; MOG: myelin oligodendrocyte glycoprotein; ON: optic neuritis; VA: visual acuity; TM: transverse myelitis; +/−: with or without.

	Spanish Study [76] Sepulveda et al., 2016; *n* = 56	German Study [60] Jarius et al., 2016; *n* = 50
**MOG assay**	CBA	CBA
**Gender (Female:Male)**	1.67:1	2.8:1
**Race**	54 Caucasian; 2 others	49 Caucasian; 1 Asian
**Median age disease onset (Range)**	37 years (18–70)	31 years (6–70)
**Clinical syndrome at onset (N)**	ON (34) LETM (12)/TM (1) ON + TM (5) ADEM (3) Brainstem syndrome (1)	ON (32) TM (9) ON + transverse myelitis (5) ADEM (3) Brainstem syndrome (1)
**Median time to second attack**	No data	5 month
**Clinical syndrome at last FU (N)**	ON (27) Recurrent (21); CRION (3/21); BL simultaneous (1/21) Monophasic (6); BL simultaneous (5/6) LETM (7)/TM (3) LETM (7); Relapsing (1) SSTM (3); Relapsing (3) ON + TM (1) ADEM (2): Monophasic MDEM (1): Relapse at 3 and 4 years Relapsing brainstem syndrome (1) NMOSD (14) Relapsing (12): ON, TM, brainstem relapse, ON + TM (5) Monophasic (2)	ON (22) Recurrent (14) Monophasic (8) TM (6) Multifocal: ON+ transverse myelitis +/− brain +/− brainstem (22)
**Relapsing% versus Monophasic %**	71% versus 29%	80% versus 20%
**Median follow-up (range), months**	43 (4–554) months	75 (1–507) months
**MOG titers, serum (range)** **MOG titers, CSF**	1/960 (1/160–1/10240) No data	1/160–1/20480 1/2–1/64
**CSF** **Cells** **OCB**	Mean= 41 (SD = 70) 3/53	Median = 33 (IQR 13–125) 6/45
**Relapse number**	125 85 ON 29 TM 2 ON+TM 5 brain 4 brainstem	205 ON 73 TM 3 cerebellum 9 brain 20 brainstem
**Annualized relapse rate**	1.11	0.83
**Outcome**	**Data on VA in 46 patients** VA < 20/100 in 19% (8/46) ON patients **Last EDSS 2 (0–7)** 0–2.5: 71% 3–3.5: 18% 4–5.5: 7% ≥6: 4%	**Data on VA in 38 patients** VA ≤ 20/100; 10/38 (26.3%) Functional blindness* in at least one eye Severe visual loss at least one eye 20/100 < VA ≤ 20/40; 4/38 (10.5%) Moderate visual loss at least one eye 20/40 < VA < 20/25; 2/38 (5.3%) Mild visual loss at least one eye 20/25 < VA < 20/20; 5/38 (13.2%) Last EDSS** (1–10) Median 3 (*n* = 40 relapsing cohort) Median 2.5 (*n* =47 total cohort) **Data on motor deficit available for 28 TM patients** Severe paresis (BMRC*** ≤ 2): 1/28 or 3.6% Moderate paresis: 4/28 or 14.3% Mild paresis: 6/28 or 21.4% Impaired ambulation (paresis + ataxia): 25%
**Concomitant autoimmune antibodies** **Concomitant autoimmune disorders**	No data *n* = 7 (13%)	*n* = 19/45 (42.2%) *n* = 4/47 (8.5%)
**Relapse management**	No detailed data Corticosteroids	122 relapses treated with IVMP Almost-complete recovery: 50% Partial recovery: 44.3% Almost no recovery: 5.7%
**Maintenance Therapy** **Medications**	46% No data	Monoclonal B-cell therapies No data

**Table 4:** Summary of the 2-case series on ADEM/MDEM followed by optic neuritis. ADEM: Acute disseminated encephalomyelitis; AQP4: Aquaporin4; GA: Glatiramer acetate; INF-beta: interferon beta; IVIG: Intravenous immunoglobulins; MDEM: Multiphasic disseminated encephalomyelitis; MOG: Myelin oligodendrocyte; ND: No data; OCB: Oligoclonal bands; ON: Optic neuritis; PLEX: Plasma exchange; TDL: Tumefactive demyelinating lesions.

	Hupke et al., 2012 [188]	Baumann et al., 2016 [191]
**N of patients**	7	8
**Median Age (range) years**	6 (4–8)	3 (1–7)
**Gender (F/M)**	6/1	5/3
**Clinical presentation** **ADEM** **MDEM** **ON**	*n* = 7 *n* = 3/7 Unilateral in *n* = 6; Bilateral in *n* = 1 Always following ADEM/MDEM N of attacks: 1–7 Inter-attack intervals: 3 weeks-2 years	*n* = 7 Unilateral/Bilateral: ND; *n* = 2
**Median inter-attack interval**	Minimum 4 weeks for ON	4 months for MDEM
**Preceding febrile illness; N; (weeks prior)**	ND	Yes; *n* = 4; 4 weeks prior
**Median follow up (range) years**	6 for *n* = 4/7	4 (1–8)
**Autoantibody**	ADEM stage: MOG-antibody (+) 3/7 and ND 4/7 MOG- antibody (+) with ON AQP4-antibody (-)	MDEM stage: MOG- antibody (+) 8/8 AQP4-antibody (-)
**CSF**	Pleocytosis Negative OCB	Pleocytosis Negative OCB in *n* = 7/8
**MRI Brain** **MRI spinal cord**	Classical ADEM findings New lesions with MDEM No new brain MRI lesions during ON ND	Classical ADEM findings with TDL and cortical GM lesions. New lesions with MDEM LETM *n* = 2 SSTM *n* =2
**Treatment**	Improvement with corticosteroids Azathioprine: Partial effectiveness IFN-beta and GA: Not effective	Corticosteroids during attacks (*n* = 8) IVIG (*n* = 1) during attack PLEX (*n* = 1) during attack IVIG Monthly (*n* = 4)
**Outcome**	Minimal to no relapses, *n* = 2 Continuous relapses, *n* = 2 Mild vision loss, *n* = 4	Normal *n* = 4 Mild-moderate deficit *n* = 4 (psychomotor and/or seizures)

**Table 5:** Summary of Overlap Syndrome or NMOSD-Encephalitis complex reported in the literature. Ab: Antibodies; ADEM: acute disseminated encephalomyelitis; CEL; contrast-enhancing lesions; CSF: cerebral spinal fluid; HSV: herpes simplex virus; EDSS: expanded disability status scale; INO: internuclear opthalmoplegia; IV: intravenous; IVIG: intravenous immunoglobulins; LETM: longitudinally extensive transverse myelitis; MOG: myelin oligodendrocyte glycoprotein; MRI: Magnetic resonance imaging; ND: no data; NL: normal; NMDAR antibody: N-Methyl-D-Asparate receptor antibody; NMO: neuromyelitis optica; OCB: oligoclonal bands; ON: optic neuritis; PLEX: plasma exchange; PRES: posterior reversible encephalopathy syndrome; TM: transverse myelitis; VGKC: Voltage-gated potassium channel; − : negative; + : positive; ( ): number of patients; * Multi-modality therapy: combination therapy of IVIG, steroids, and plasma exchange * CT scan includes imaging of the abdomen, chest, and pelvis to rule out malignancy.

	Demographics	Presentation	MRI	Treatment/Response	Serum Ab (Number of Patients)	CSF/an Cillary Testing (Number of Patients)	Final Outcome
**Kruer et al., 2010** [193]	*n* = 1 F, 15 years	Seizures, encephalopathy, movement disorder, myelopathy, unilateral ON	Multifocal CEL; LETM	Prednisone: improvement. Interferon-B therapy, cyclophosphamide: failed therapy Multi-modality therapy *: drastic improvement	AQP4: -	Pleocytosis: + Protein: 2x NL Glucose: low OCB: + NMDAR: + Pan CT scan: -	Asymptomatic
**Lekoubou et al., 2012** [198]	*n* = 1 F, 34 years	Encephalopathy, movement disorder, myelopathy	Widespread, multifocal white matter lesions, right frontal contrast enhancing lesion, LETM	IVIG, steroids: failed therapy Immunosuppressant: dramatic improvement	AQP4: -	Pleocytosis: + Protein: elevation Glucose: low OCB: + NMDAR: + Viral panel: - Pan CT scan: -	Minor residual cognitive impairment
**Zoccarato et al., 2013** [199]	*n* = 1 F, 50 years	Encephalopathy, movement disorder, myelopathy, unilateral ON	T2 hyperintense lesions in cortical medial temporal lobe, pons, hypothalamus, medulla, cervical & dorsal spine	Hystero-adnexectomy, oral steroids: improvement PLEX, steroids: improvement Immunosuppressant: stable disease	AQP4: + NMDAR: +	Pleocytosis: ND Protein: ND Glucose: ND OCB: + NMDAR: + EEG: - Pan CT scan: ovarian teratoma	Stable disease
**Outteryck et al., 2013** [200]	*n* = 1 F, 65 years	Encephalopathy, movement disorder, myelopathy, subclinical unilateral ON	LETM with gadolinium enhancement, T2 hyperintensities in insular regions, medial temporal lobes & thalamus, gadolinium enhancement in meninges & ventricles	IV steroids: paraparesis worsened PLEX, immunosuppressant, oral steroids: significant improvement	AQP4: - NMDAR: +	Pleocytosis: + Protein: elevation Glucose: ND OCB: + NMDAR: + Pan CT scan: -	Death: due to rapidly evolving pneumocystis pneumonia
**Hacohen et al., 2014** [194]	*n* = 15 M (6), 3–15 years F (9), 2–15 years	Seizures, encephalopathy, movement disorder, myelopathy, unilateral/bilateral ON	Normal, optic nerve signal changes, multiple white matter abnormalities, spinal cord involvement, periventricular/juxtacortical, brainstem lesions	ND	AQP4: +(3) NMDAR: +(2) MOG: +(7) VGKC: +(3) GlyR: +(1)	Pleocytosis: + (3) Protein: ND Glucose: ND OCB: +(3)/-(7)/ND (5) NMDAR: ND Viral panel: ND MOG: ND EEG: ND Pan CT scan: ND	Full recovery (4), EDSS 1 (6), EDSS 3 (2), EDSS 4 (2), EDSS 7 (1), seizures (2), Visual loss (1)
**Hacohen et al., 2014** [195]	*n* = 10 M (5), 2–18 years F (5), 1.33–11 years	Brainstem encephalitis HSV encephalitis Unilateral/bilateral ON ADEM Recurrent hyperventilation, dizziness, double vision	Normal (2), brainstem signal change (2), white matter changes (7), multiple cranial nerve enhancement (1), gray matter changes (1)	No treatment: (1) Multimodality treatment with IV, oral steroids, IVIG and immunosuppressants	AQP4: ND NMDAR: +(10) MOG: ND VGKC: ND GlyR: ND	Pleocytosis: ND Protein: ND Glucose: NDOCB: +(1)/-(2)/ ND (7) NMDAR: +(4)/ ND (6) MOG: + (3)/ND (7)	Full recovery (4) Incomplete (5) Unchanged (1)
**Titulaer et al., 2014** [197]	*n* = 12 M (5), 10–38 years F (7), 8–55 years	Seizures, encephalopathy, movement disorder, myelopathy (LETM) unilateral/bilateral ON	ND (2), Normal (1), optic nerve signal changes (1), white matter lesions (4), T2/FLAIR abnormalities in various aspects of the brain (7), spinal cord involvement (4), Gd enhanced lesions (6), brainstem lesions (4)	No treatment: (1) Multimodality treatment with steroids, IVIG, PLEX, interferon, imunosuppressanT	AQP4: +(3), -(5), ND (4) NMDAR: +(2), -(5), ND (5) MOG: +(6), -(3), ND (3) VGKC: ND GlyR: ND	Pleocytosis: +(8)/-(4) Protein and glucose ND OCB: +(5)/-(5)/ND (2) NMDAR: +(12) MOG: +(7)/-(4)/ND (1)	ND
**Splendiani et al., 2016** [196]	*n* = 1 M, 17 years	Seizures, encephalopathy, movement disorder, myelopathy	T2 & FLAIR cortical-subcortical hyperintensities in right cerebellar hemisphere, ipsilateral cerebellar tonsil, with faint T1 hypointensity	Olanzapine: progression of symptoms IV steroids: gradual improvement	AQP4: ND NMDAR: + MOG: ND VGKC: ND GlyR: ND	CSF findings: ND NMDAR: + Viral panel: - EEG: - Pan CT scan: -	Asymptomatic
**Ogawa et al., 2017** [192]	*n* = 4 patients M, 23–39 years	Seizures, encephalopathy, ON	T2 & FLAIR unilateral cortical hyperintensities; no ADEM-like lesions.	IV steroids with significant improvement 2–3 antiepileptics/patient	AQP4: - MOG: +(3) NMDAR: -(4) AMPA: -(4) LGI1: -(4) CASPR2: -(4) GABAb: -(4)	Pleocytosis + Protein: NL-1.5xNL OCB: ND MBP: NL (3); ND (1) MOG: +(3); ND (1)	Remain on antiepileptics Otherwise full recovery

**Table 6:** Maintenance therapy for NMOSD [233,234]. GI: Gastrointestinal; UTI: urinary tract infection; URI: upper respiratory infection; PML: progressive multifocal leukoencephalopathy; DVT: deep venous thrombosis; TB: tuberculosis.

Medication Name	Mechanism of Action (MOA)	Dosage	Treatment Response	Side Effects
**Azathioprine**	Thiopurine antagonist of endogenous purines in DNA and RNA, interferes with lymphocyte proliferation	Initial: 2–3 mg/kg/day with concomitant prednisone (5–60 mg daily) for 6–12 months Maintenance: 2–3 mg/kg/day	Approximately 50/50 chance of preventing additional relapse	Nausea, diarrhea, rash, recurrent infections, leukopenia, transaminase elevation, increased risk of lymphoma
**Cyclophosphamide**	Cytotoxic alkylating agent, inhibits mitosis	Initial: 1000 mg every 2 months with associated steroid Maintenance: same as initial dosing	Specific treatment response unavailable – only recommended when other immunosuppressive therapies fail or are not available due to contradictory preliminary findings.	GI symptoms, hyponatremia, heart block, pancytopenia, opportunistic infections
**Eculizumab**	Binds to the complement protein C5 specifically, inhibiting its cleavage to C5a and C5b and subsequent generation of the terminal complement complex C5b-9	Standard dose: IV 600 mg weekly for four weeks, then IV 900 mg every two weeks	Specific treatment response unavailable at this time	Headache, increased risk of infection with encapsulated organisms, especially meningococcal infections
**Methotrexate**	Folic acid antagonist	Initial: start with 7.5 mg weekly with upward titration and concomitant prednisone (5–60 mg daily) Maintenance: 7.5–15 mg weekly with concurrent prednisone (5–10 mg daily for at least sixmonths)	Remission rates in up to 2/3 of subjects when used as monotherapy or in conjunction with corticosteroids	Pneumonitis, GI upset, cytopenia, hepatotoxicity
**Mitoxatrone**	Causes DNA cross-linking and strand breaks, interferes with DNA repair	Initial: 12 mg/m² for 3–6 months Maintenance: 6–12 mg/m² every 3 months	Remission in up to 70% of subjects when dosed appropriately	Nausea, transaminase elevation, leukopenia, hair loss, amenorrhea, minor infections including UTI and URI, rarely heart failure and acute leukemia
**Mycophenolate mofetil**	Inhibits inosine monophosphate dehydrogenase, impairs B- and T-cell synthesis	Initial: 1000–2000 mg daily with concurrent prednisone (5–60 mg daily) Maintenance: 1000–2000 mg	Approximately 60–75% achieve remission with fewer side effects and adverse effects	Photosensitivity, recurrent infections, headache, constipation, abdominal pain, leukopenia, PML is rare
**Rituximab**	Removal of B cells as antigen presenting cells and reduction in the CD20+ early plasmablast population generating anti-quaporin-4 antibodies	Initial: 1000 mg weekly for two weeks or 375 mg/m² weekly for four weeks Maintenance: 375 mg/m² or 1000 mg weekly for 2 weeks when CD19 count >1% on flow cytometry	Remission rates up to 83% were achieved with persistent B cell depletion	Sepsis, infections (Herpes zoster, UTIs, URIs), leukopenia, transaminase elevation, PML is rare
**Tocilizumab**	Directed against the IL-6 receptor reducing plasmablast survival, inhibiting AQP4 antibody production	Standard dose: 8 mg/kg every four weeks	Specific treatment response unavailable at this time	GI disturbance, fatigue, UTIs, neutropenia, leukopenia, elevation of cholesterol, transient mild transaminase elevation, DVT, TB reactivation

## Supplementary Materials

The following are available online at www.mdpi.com/link/2076-3425/7/10/138/s1, Table S1: Evolution of MRI diagnostic criteria for dissemination in time (DIT) and dissemination in space (DIS). CEL: Contrast-enhancing lesion, Table S2: Brain Imaging Findings in Neuromyelitis Optica Spectrum Disorder, Figure S1: 50-year-old female, with seronegative NMO and cloudlike enhancement on axial T1 with contrast enhancement (**1a**). 55-year-old African American female, with AQP4-NMOSD; presence of an ovoid right frontal juxtacortical/subcortical T2 hyperintensity (**1b**) with cloud like enhancement on axial T1 with contrast (**1c**). Repeat MRI of the brain 6 months later showed a significant improvement in T2 hyperintensity (**1d**) underlining the evanescent nature of NMOSD lesions, Figure S2: Axial FLAIR cuts (2a and 2c) demonstrate a right middle cerebellar peduncle and midbrain lesions with leptomeningeal contrast enhancement on T1 with contrast (2b and 2d) in a 50-year-old female with seronegative NMOSD, Figure S3: Sagittal and axial FLAIR MRI of the brain demonstrate diffuse involvement and swelling of the corpus callosum (3a and 3c) with high intensity rim and lower intensity core. Axial T1 with contrast demonstrates heterogeneous contrast enhancement. Repeat brain MRI (3b), 8 months later, demonstrates resolved edematous state with some callosal atrophy. Figure S4: 40-year-old Caucasian women presenting with intractable hiccups, nausea and vomiting and a dorsal brainstem lesion with a linear component involving the medulla and cervicomedullary junction seen on sagittal STIR (4a) and enhancing with contrast on T1. Aquaporin 4 antibody was positive. Figure S5: 43-year-old female, with AQP4-NMOSD; axial FLAIR demonstrates non-specific white matter lesions, (5a) a periventricular lesion around the posterior horn of the left lateral ventricle (5b), confirmed on sagittal FLAIR (5c).

## Abbreviations

The following abbreviations are used in this manuscript:

ADEM	Acute Disseminated Encephalomyelitis
ADEM-ON	Acute Disseminated Encephalomyelitis-Optic Neuritis
ATM	Acute Transverse Myelitis
APTM	Acute Partial Transverse Myelitis
ACTM	Acute complete Transverse Myelitis
CRION	Chronic Relapsing Inflammatory Neuropathy
GFAP	Glial Fibrillary Acid Protein
LETM	Longitudinally Extensive Transverse Myelitis
MS	Multiple Sclerosis
MS-ON	MS-Associated Optic Neuritis
MS-TM	MS-Asociated Transverse Myelitis
MS-BS	MS-Asociated Brainstem Syndrome
MOG	Myelin Oligodendrocyte Glycoprotein
MOG-ON	Myelin Oligodendrocyte Glycoprotein- Associated Optic Neuritis
MOG-TM	Myelin Oligodendrocyte Glycoprotein- Associated Transverse Myelitis
MDEM-ON	Multiphasic Disseminated Encephalomyelitis-Optic Neuritis
MDEM-ON	Multiphasic Disseminated Encephalomyelitis-Optic Neuritis
APQ4	aquaporin 4
NMOSD	Neuromyelitis Optica Spectrum Disorder
NMOSD-ON	Neuromyelitis Optica Spectrum Disorder-Associated Optic Neuritis
NMOSD-TM	Neuromyelitis Optica Spectrum Disorder-Associated Transverse Myelitis Optic Neuritis
NMOSD-BS	Neuromyelitis Optica Spectrum Disorder-Associated Brainstem Syndrome
NMDA-R	N-Methyl-D-Aspartate Receptor
ON	Optic Neuritis
SION	Single Inflammatory or Isolated Optic Neuritis
RION	Recurrent Inflammatory or Isolated Optic Neuritis
SSTM	Short Segment Transverse Myelitis
