# Supplementary material for: Pattern Recognition of the Multiple Sclerosis Syndrome
Source: Brain Sci. 2017 Oct 24;7(10):138. doi: 10.3390/brainsci7100138 (PMC5664065; doi:10.3390/brainsci7100138)

Supplementary Materials

McDonald Criteria 2001 [8]	Revised McDonald Criteria 2005 [10]	Revised McDonald Criteria 2010 [9]	MAGNIMS Criteria 2016 [11]
DIS can be demonstrated by having three out of four of the following:<br>1. One CEL or 9 T2-hyperintense lesions if there is no CEL<br>2. At least one infratentorial lesion<br>3. At least one juxtacortical lesion<br>4. At least three periventricular lesions	DIS can be demonstrated by having three out of four of the following:<br>1. ≥ 1 CEL or 9 T2-hyperintense lesions if there is no CEL<br>2. ≥ 1infratentorial lesion<br>3. ≥ 1juxtacortical lesion<br>4. ≥ 3 periventricular lesions	DIS can be demonstrated by ≥ 1 T2 lesion in at least two of the four areas of the CNS:<br>1. Periventricular<br>2. Juxtacortical<br>3. Infratentorial<br>4. Spinal cord	DIS can be demonstrated by having at least two out of five of the following:<br>1. ≥ 3 periventricular lesions<br>2. ≥ 1 infratentorial lesion<br>3. ≥ 1 spinal cord lesion<br>4. ≥ 1 optic nerve lesion<br>5. ≥ 1 cortical or juxtacortical lesion
Note: One spinal cord lesion can substitute for vone brain lesion.	*Note:* A spinal cord is equivalent to an infratentorial lesion and can contribute with brain lesions to the required number of T2 lesions. CEL spinal cord lesion is considered equivalent to a brain CEL	*Note:* A CEL is not required for DIS. If a subject has a brainstem or spinal cord syndrome, the symptomatic lesions are excluded from the criteria and do not contribute to the lesion count.	*Note:* If a patient has a brainstem or spinal cord syndrome, or optic neuritis, the symptomatic lesion(s) is not excluded from the criteria and can contribute to the lesion count.
DIT can be demonstrated by the following manner:<br>1. If a first scan occurs ≥ 3 months after the onset of the clinical event, the presence of a CEL is sufficient to demonstrate DIT, provided that it is not at the site implicated in the original clinical event. If there is no CEL, a follow-up scan is required 3 months later. A new T2 or CEL at this time then fulfills the criteria for DIT. If the first scan is performed < 3 months after the onset of the clinical event, and a second scan performed three months or longer after the clinical event shows a new CEL, then this provides sufficient evidence for DIT. If no enhancing lesion is seen at this second scan, a further scan, not before 3 months after the first scan that shows a new T2 lesions or a CEL, will suffice.	DIT can be demonstrated by the following two ways using imaging:<br>1. Detection of CEL at least three months after the onset of the initial clinical event, if not at the site corresponding to the initial event.<br>2. Detection of a new T2 lesion if it appears at any time compared with a reference scan done at least 30 days after the onset of the initial clinical event.	DIT can be demonstrated by the following manner:<br>1. A new T2 and/or CEL(s) on follow-up MRI, with reference to a baseline scan, irrespective of the timing of the baseline MRI.<br>2. Simultaneous presence of asymptomatic CEL and non-CEL at any time.	DIT can be demonstrated by the following manner:<br>1. A new T2 and/or CEL(s) on follow-up MRI, with reference to a baseline scan, irrespective of the timing of the baseline MRI.<br>2. Simultaneous presence of asymptomatic CEL and non-CEL at any time.

Table S1: Evolution of MRI diagnostic criteria for dissemination in time (DIT) and dissemination in space (DIS). CEL: Contrast-enhancing lesion.

MRI Abnormality	Descriptors and Findings
**Contrast enhancement (Described in 9-36% of NMOSD) [18-20, 23-27]**	• Cloud-Like (figures 1b & d)<br>o More common type<br>o Subtle parenchymal, patchy, heterogeneous<br>• Linear periependymal or "pencil thin" lesions<br>o More characteristic type<br>o Reflective of AQP4-rich areas<br>o T2 hyperintensity might be present, spindle like<br>• Leptomeningeal enhancement (figures 2b & d)<br>o Rare; reflective to bindings of AQP4 antibody to AQP4 in the pial and subpial space<br>o Linear, thick and extensive; supra- or infratentorial<br>• Ring and open ring enhancement<br>o Rare in NMOSD<br>o Seen with seronegative NMOSD
**Periependymal lesions [23-25, 29]**	• Corpus callosum lesions (figures 3a-d)<br>o Multiple, edematous, heterogenous, and along ependymal lining<br>o Possible extension into the cerebral hemispheres<br>o Possible cognitive dysfunction<br>o Higher intensity rim and lower intensity core (marbled appearance)<br>o Diffuse involvement and swelling of splenium may exist acutely<br>▪ Disappear with time<br>▪ Chronic cystic lesion in genu<br>▪ Severe atrophy<br>• Diencephalic lesions<br>o Thalamus, hypothalamus, anterior border midbrain<br>o Asymptomatic, SIADH, narcolepsy or endocrine abnormalities<br>• Dorsal brainstem lesions: very specific of NMOSD (figures 4a & b)<br>o Area postrema and nucleus tractus solitaries<br>o Frequent contiguity with cervical cord lesions<br>o Intractable hiccups, nausea, and vomiting<br>o Early presentation as linear lesions<br>o Can extend to the medulla
**Hemispheric Lesions [18-20, 23-27]**	• Tumefactive > 3 cm in longest diameter, confluent, heterogeneous, spindle-like or radial shaped<br>o Evanescent (mechanism: astrocytopathy or intramyelinic edema)<br>o Facilitated diffusion<br>• Occasionally mimic posterior reversible encephalopathy syndrome
**Corticospinal Tract(s) [23-24]**	• Longitudinally extensive lesions from internal capsule to pons<br>• Significance unclear as the location is not associated to AQP4-rich structures
**Non-specific lesions [23-24]**	• Unexplained and silent<br>• Deep or subcortical white matter<br>• MS-like in 10-12% and occasionally fulfilling Barkhof's criteria (5b & c)<br>• Punctate or < 3 mm (figure 5a)
**Cortical lesions [27, 155-156)**	• Absent but selective decrease in cortical thickness<br>• Rare, associated with leptomeningeal enhancement suboptimally treated patients<br>• Recently associated with MOG-NMOSD (6a-f)<br>• Ultra-high field MRI might improve detection of cortical lesions in NMOSD

Table S2: Brain Imaging Findings in Neuromyelitis Optica Spectrum Disorder

Figure S1: 50-year-old female, with seronegative NMO and cloudlike enhancement on axial T1 with contrast enhancement (**1a**). 55-year-old African American female, with AQP4-NMOSD; presence of an ovoid right frontal juxtacortical/subcortical T2 hyperintensity (**1b**) with cloud like enhancement on axial T1 with contrast (**1c**). Repeat MRI of the brain 6 months later showed a significant improvement in T2 hyperintensity (**1d**) underlining the evanescent nature of NMOSD lesions.

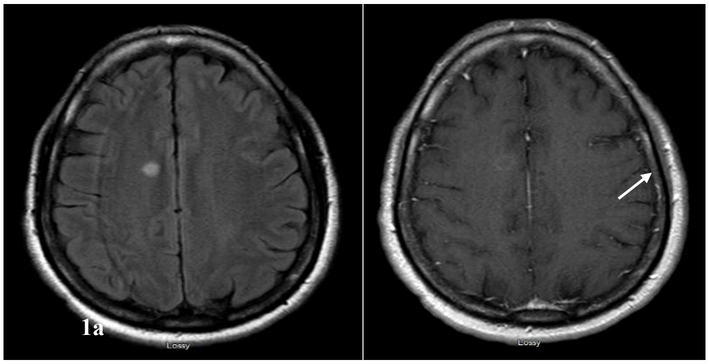

Figure S2: Axial FLAIR cuts (**2a** and **2c**) demonstrate a right middle cerebellar peduncle and midbrain lesions with leptomeningeal contrast enhancement on T1 with contrast (**2b** and **2d**) in a 50-year-old female with seronegative NMOSD.

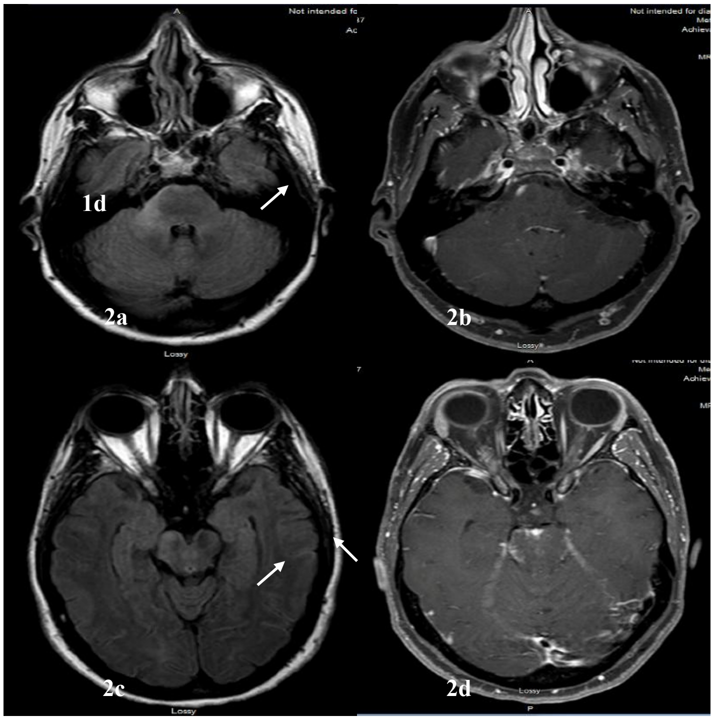

Figure S3: Sagittal and axial FLAIR MRI of the brain demonstrate diffuse involvement and swelling of the corpus callosum (**3a** and **3c**) with high intensity rim and lower intensity core. Axial T1 with contrast demonstrates heterogeneous contrast enhancement. Repeat brain MRI (**3b**), 8 months later, demonstrates resolved edematous state with some callosal atrophy.

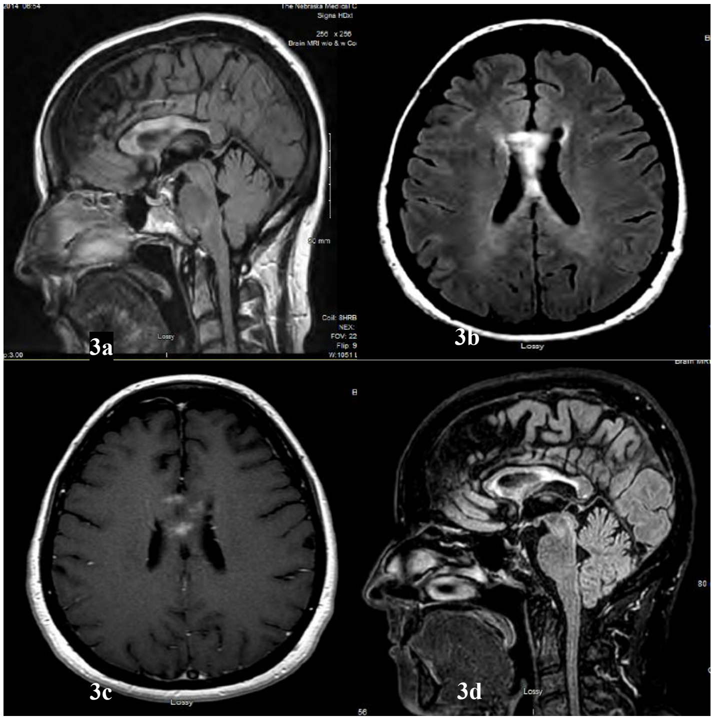

Figure S4: 40-year-old Caucasian women presenting with intractable hiccups, nausea and vomiting and a dorsal brainstem lesion with a linear component involving the medulla and cervicomedullary junction seen on sagittal STIR (**4a**) and enhancing with contrast on T1. Aquaporin 4 antibody was positive.

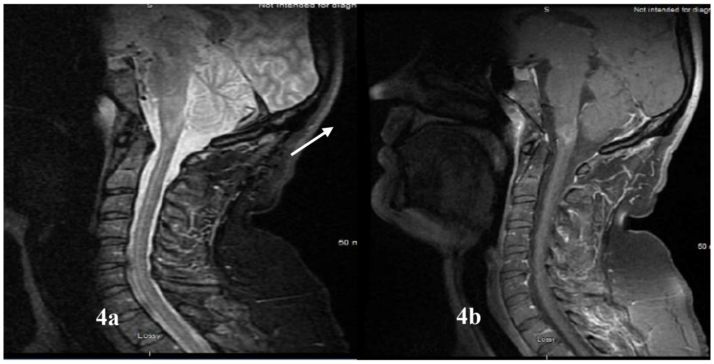

Figure S5: 43-year-old female, with AQP4-NMOSD; axial FLAIR demonstrates non-specific white matter lesions, (**5a**) a periventricular lesion around the posterior horn of the left lateral ventricle (**5b**), confirmed on sagittal FLAIR (**5c**).

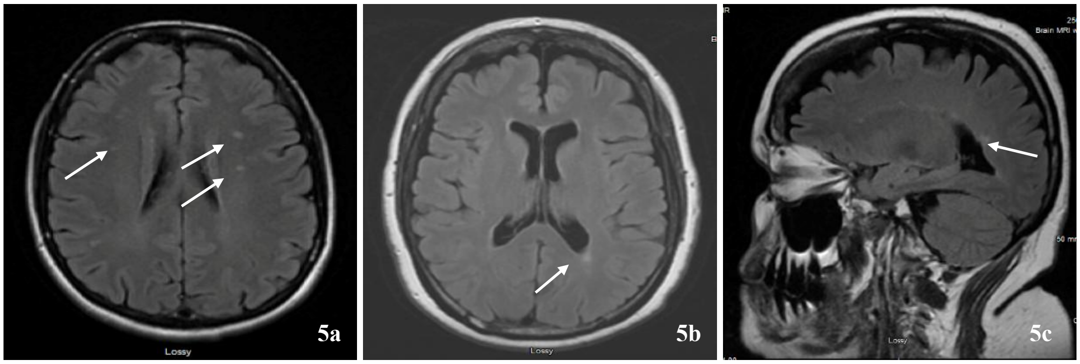

Supplement: Supplementary file 1 [file brainsci-07-00138-s001.pdf]
